# Synthesis, Characterization and In Vitro Evaluation of Hybrid Monomeric Peptides Suited for Multimodal Imaging by PET/OI: Extending the Concept of Charge—Cell Binding Correlation

**DOI:** 10.3390/ph14100989

**Published:** 2021-09-28

**Authors:** Marco Maspero, Xia Cheng, Valeska von Kiedrowski, Clelia Dallanoce, Björn Wängler, Ralph Hübner, Carmen Wängler

**Affiliations:** 1 Biomedical Chemistry, Clinic of Radiology and Nuclear Medicine, Medical Faculty Mannheim, Heidelberg University, Theodor-Kutzer-Ufer 1-3, 68167 Mannheim, Germany; marco.maspero@medma.uni-heidelberg.de; 2 Medicinal Chemistry Section “Pietro Pratesi”, Department of Pharmaceutical Sciences, University of Milan, Via L. Mangiagalli 25, 20133 Milan, Italy; clelia.dallanoce@unimi.it; 3 Molecular Imaging and Radiochemistry, Clinic of Radiology and Nuclear Medicine, Medical Faculty Mannheim, Heidelberg University, Theodor-Kutzer-Ufer 1-3, 68167 Mannheim, Germany; Xia.Cheng@medma.uni-heidelberg.de (X.C.); Valeska.vonKiedrowski@medma.uni-heidelberg.de (V.v.K.); Bjoern.Waengler@medma.uni-heidelberg.de (B.W.)

**Keywords:** multimodal imaging, PET/OI, GRPR, MC1R, α_v_β_3_, receptor affinity, ^68^Ga, fluorescent dyes

## Abstract

In the context of hybrid multimodal imaging agents for gastrin releasing peptide receptor (GRPR) targeting, a correlation between the net charge and the receptor affinity of the agents was recently found. In particular, a decrease in in vitro GRPR binding affinity was observed in case of an increasing number of negative charges for dually labeled GRPR-specific peptide dimers suited for positron emission tomography and optical imaging (PET/OI). This adverse influence of anionic charges could be in part compensated by a higher valency of peptide multimerization. However, it remains unknown whether this adverse effect of anionic charges is limited to peptide multimers or if it is also found or even more pronounced when GRPR-specific peptide monomers are dually labeled with fluorescent dye and chelating agent/radionuclide. Moreover, it would be important to know if this effect is limited to GRPR-specific agents only or if these observations also apply to other dually labeled peptides binding to other receptor types. To address these questions, we synthesized hybrid labels, comprising a chelator, different fluorescent dyes carrying different net charges and a functional group for bioconjugation and introduced them into different peptides, specifically targeting the GRPR, the melanocortin-1 receptor (MC1R) and integrin α_v_β_3_. The synthesized conjugates were evaluated with regard to their chemical, radiochemical, photophysical and receptor affinity properties. It was found that neither the ^68^Ga-radiolabeling nor the fluorescence characteristics of the dyes were altered by the conjugation of the MIUs to the peptides. Further, it was confirmed that the net number of anionic charges has a negative effect on the GRPR-binding affinity of the GRPR-targeting MIU-peptide monomer conjugates and that this same effect was also found to the same extent for the other receptor systems studied.

## 1. Introduction

Molecular imaging is defined as a non-invasive method which allows the visualization, characterization, and measurement of biological processes at molecular and cellular levels in living systems [1]. Its application in clinical oncology has undergone an explosive growth in the past few decades [2], since it can potentially provide detailed information about tumor characteristics, location, dimension, dispersion to lymph nodes and staging [3]. As a result, molecular imaging is crucial to achieve a correct tumor diagnosis and to enable effective therapy, resulting in better outcome for oncological patients. A variety of imaging modalities are available [4,5,6,7] and each of these techniques presents its own particular strengths, as well as intrinsic drawbacks. PET, for example, is a well-established clinical tool for whole-body and target-specific imaging [2,5,8], for which radiolabeled molecular probes are used for the highly specific and sensitive identification and localization of tumors. However, it cannot assist the surgeon during surgery in safely identifying tumor margins, bystanding small metastases and malignantly transformed lymph nodes. A possible solution is the application of OI for surgical navigation [9,10]. This technique uses non-ionizing radiation to illuminate endogenous or exogenous sources of contrast, and enables real-time, high resolution molecular image-guided surgery. Despite the high spatial resolution and the real-time imaging capability, the lack of penetration depth due to tissue scattering and absorption of light prevents its use for whole body imaging. Therefore, great interest derived from the possibility to combine PET and OI in a multimodal imaging approach.

**Figure 1:**
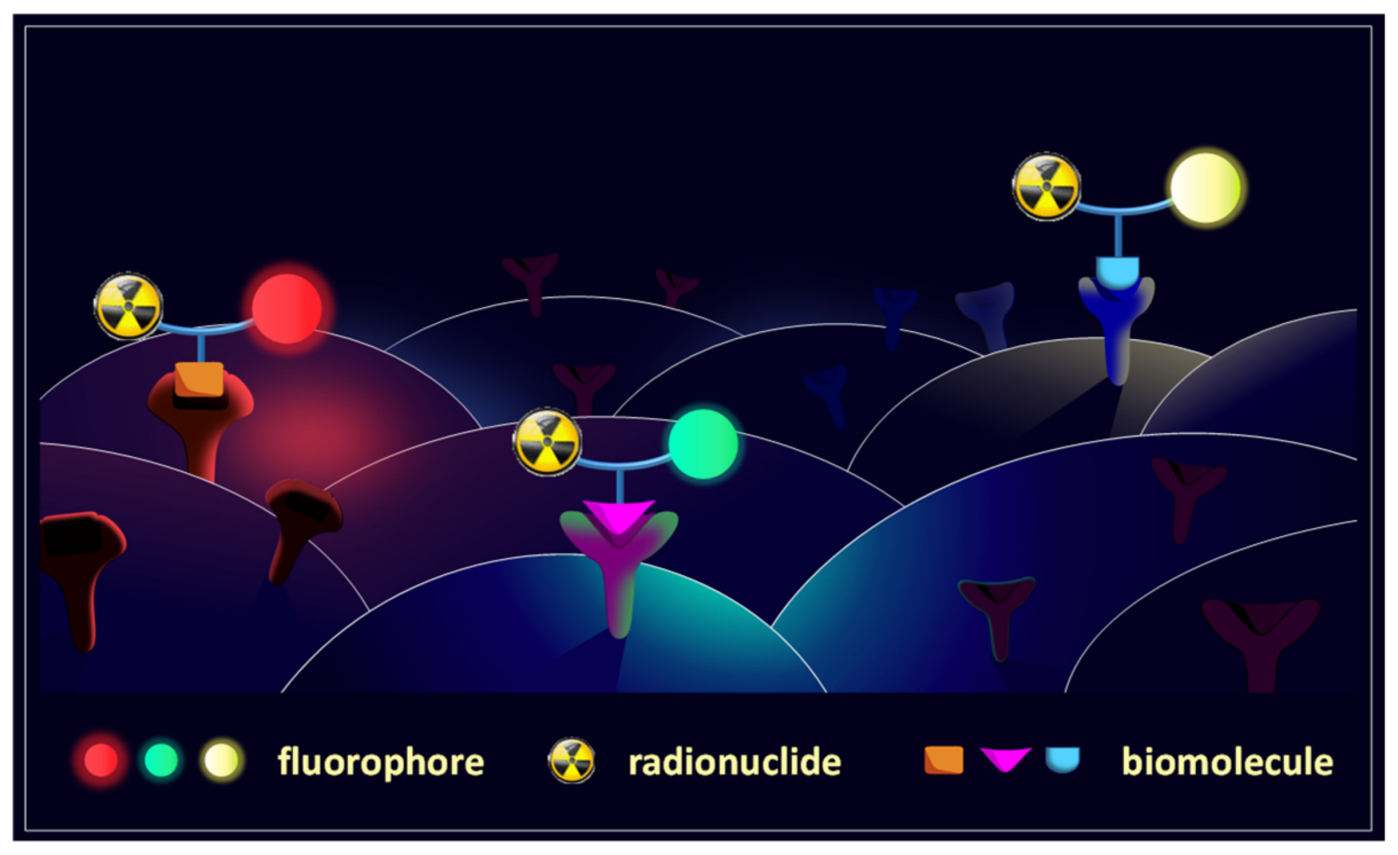
Schematic depiction of the concept of multimodal imaging agents for target-specific imaging via PET/OI. The hybrid compounds present a positron emitter (for PET imaging) and a fluorescent dye (for OI), both integrated in the structure of a target-specific biomolecule (e.g., peptide or antibody). This approach can be used to delineate a target structure by both imaging modalities and thus enables a correct and sensitive diagnosis and supports a following surgical intervention. Using different fluorescent dyes in combination furthermore enables to simultaneously detect and discriminate different target structures on the same tumor cell.

The synergistic application of two imaging modalities has increasingly become a focus in clinical research, as it allows to take advantage of the strengths of each technique, as well as to take into account their intrinsic limitations [11,12,13]. The particular combination of PET/OI exhibits a very high potential, since it enables a preoperative sensitive and target-specific total body scan by PET for the visualization of malignancies, followed by intraoperative fluorescence-guided surgery by OI for the accurate detection and thus resection of tumor borders, small metastases and infected lymph nodes [14,15,16,17]. This approach is most useful if tumor-specific multimodal imaging agents are used for the specific and sensitive visualization of the target malignancies by both modalities (Figure 1).

Combined multimodal agents comprising both reporter units is preferable to two different probes applied for PET and OI separately, since such hybrid combined agents assure equal pharmacokinetic profiles in both modalities and the visualization of the same structures by both imaging techniques. E.g., an important factor in terms of in vivo pharmacokinetics is the compound’s overall charge and charge distribution. It can influence parameters such as the interaction of the agent with blood proteins such as albumin or metabolization and clearance as well as re-absorption in the kidneys. As small molecular changes can thus considerably impact the pharmacokinetic profile of a substance, the development of two different imaging agents, one carrying a fluorescent dye and the other carrying a radionuclide, is likely to result in different in vivo pharmacokinetics of both compounds, being unfavorable for the multimodal imaging approach.

Thus, efforts were made to develop hybrid multimodal imaging probes integrating in their structure a fluorescent dye, a positron emitter and a tumor-targeting vector, designed in a way that the physical, chemical and biological properties of the agents are affected as little as possible [13,18]. For the target-specific uptake of imaging agents into malignancies, peptide-based compounds present—besides high target affinity and specific accumulation in target tissues—various advantages compared to other targeted biomolecules as antibodies such as straightforward chemical synthesis, fast pharmacokinetics and efficient tissue penetration [19].

However, due to the relatively small dimensions of peptides, the introduction of both reporter probes in different positions of the peptidic structure can easily alter their physical, chemical and biological properties, possibly compromising their ability to interact with the target. Therefore, it is optimal to develop multimodal imaging units (MIUs), composed of a structure motive enabling the introduction of a positron emitter for PET detection and a fluorescent dye, which can be easily introduced by an efficient and chemoselectively reacting functional group into a target-specific carrier molecule in only one position without altering its binding profile.

**Figure 2:**
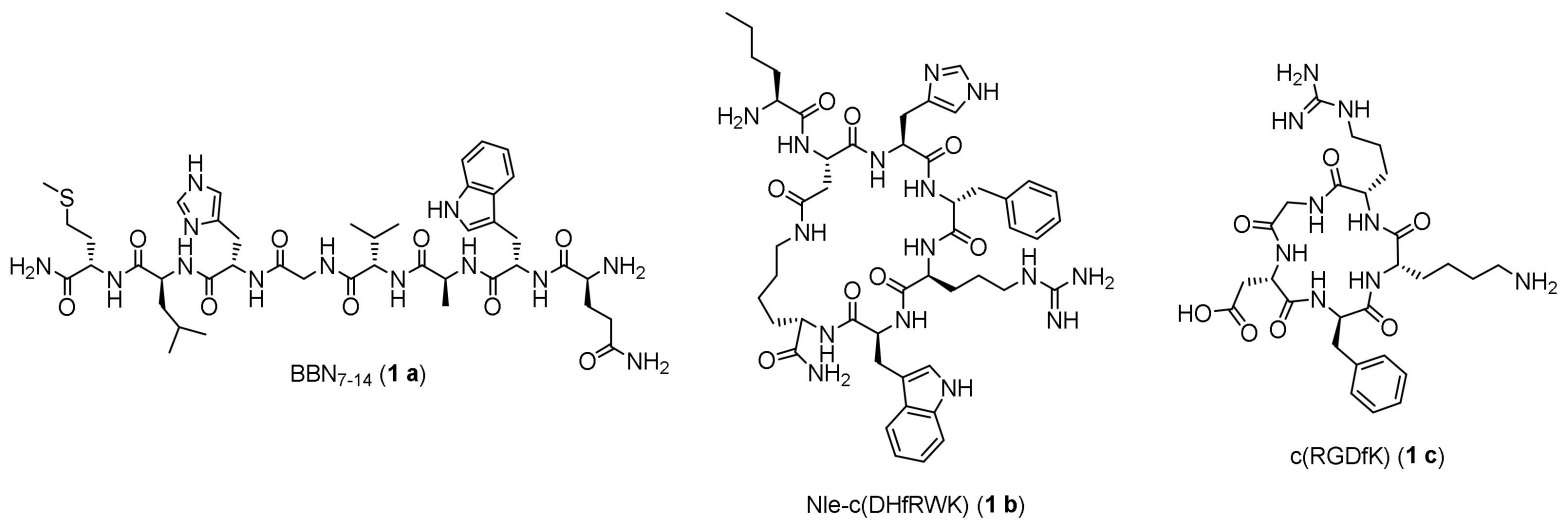
Structures of the peptides BBN_7-14_ (**1 a**), Nle-c(DHfRWK) (**1 b**) and c(RGDfK) (**1 c**), specifically binding to the GRPR, MC1R and integrin α_v_β_3_ which were chosen as receptor-specific carriers for the preparation of the hybrid, dually labelled tumor targeting vectors.

A previous successful attempt [20,21] demonstrated that it was possible to develop and insert MIUs composed of different small dye molecules and a NODA-GA chelator ((1,4,7-triazacyclononane-4,7-diyl)diacetic acid-1-glutaric acid) into the structure of a PESIN-homodimer (PESIN: PEG_3_-BBN_7-14_, BBN_7-14_ (Figure 2, **1 a**): truncated peptide sequence of the endogenous GRPR ligand bombesin), without per se altering the binding properties of the resulting conjugates.

It was observed that in the designed MIUs, neither the radiolabeling nor the photophysical properties were altered compared to the isolated components, even when conjugated to the PESIN-homodimer. However, although the interaction of the synthesized bioconjugates with the target GRPR was not considerably affected by the introduction of the chelator alone or the rather bulky chelator and dye-comprising MIUs, a strong negative correlation between the number of anionic charges carried by the respective fluorescent dye and the GRPR binding affinity of the resulting hybrid imaging agents was found. To be more specific, it was found that the higher the number of negative charges in the selected dye was, the worse the resulting binding affinity to the targeted receptor. This concept was confirmed using diverse PESIN-homodimer conjugates presenting different dye units [20,21], as well as with a PESIN-homotetramer conjugate, being characterized by a higher peptide valency [22]. In this latter case, it was interestingly observed that the adverse influence of the negatively charged synthons on the target receptor affinity was in part compensated by the higher number of peptide units in the molecule, arguing a possible intramolecular spatial shielding effect of the peptides on the adverse charges.

Taken together, these former results on MIU-modified peptide homomultimers allowed to conclude at least for the GRPR that an increasing number of anionic charges in direct vicinity to the receptor-affine peptide unit has a negative influence on the target receptor binding affinity, while the increment of peptidic binding motives in the imaging agent can partly compensate this negative effect.

However, it remains unknown whether the adverse effect of negative charges is limited to peptide multimers or if it is also found—and potentially even more pronounced—in case of dually labeled GRPR-specific peptide monomers. Regarding the observed shielding of charges by peptide strands, it was also possible that peptide monomers are even more susceptible to charge introduction then the afore-studied peptide dimers. Moreover, it would be important to know if this negative correlation between the number of anionic charges and receptor affinities is limited to GRPR-specific binders or if these observations also apply to other dually labeled peptides binding to other receptor types.

Aiming to test and verify the prevailing theory in the present study, we designed and synthesized MIU-modified monomeric GRPR binders (corresponding to the reported PESIN homodimer-based hybrid imaging agents) as well as the corresponding analogs comprising other peptides, targeting two other relevant receptors for tumor imaging: the melanocortin-1 receptor (MC1R), which is stably overexpressed on the majority of primary human malignant melanomas [23] and integrin α_v_β_3_, which is a protein being related to neo-angiogenetic processes and overexpressed in the blood vessels of many human tumors [24]. In detail, the MIUs were conjugated to the melanocyte stimulating hormone (α-MSH) derivative Nle-c(DHfRWK) (Figure 2, **1 b**) [25], addressing the melanocortin-1 receptor (MC1R) and the cyclic peptide c(RGDfK) (Figure 2, **1 c**) [26], targeting integrin α_v_β_3_. In the following, it was determined for the hybrid multimodal agents if the introduction of an increasing number of negative charges affected the chemical, radiochemical, photophysical or biological receptor affinity properties of the novel MIU-peptide-conjugates.

## 2. Results and Discussion

### 2.1. Synthesis of the Maleimide-Modified Peptide Units **3 a-c**

As outlined before, the three peptides BBN_7-14_, Nle-c(DHfRWK) and c(RGDfK) (Figure 2) were chosen to develop comparably MIU-modified dually labeled hybrid agents for multimodal imaging targeting the receptors GRPR, MC1R and integrin α_v_β_3_.

**Scheme 1:**
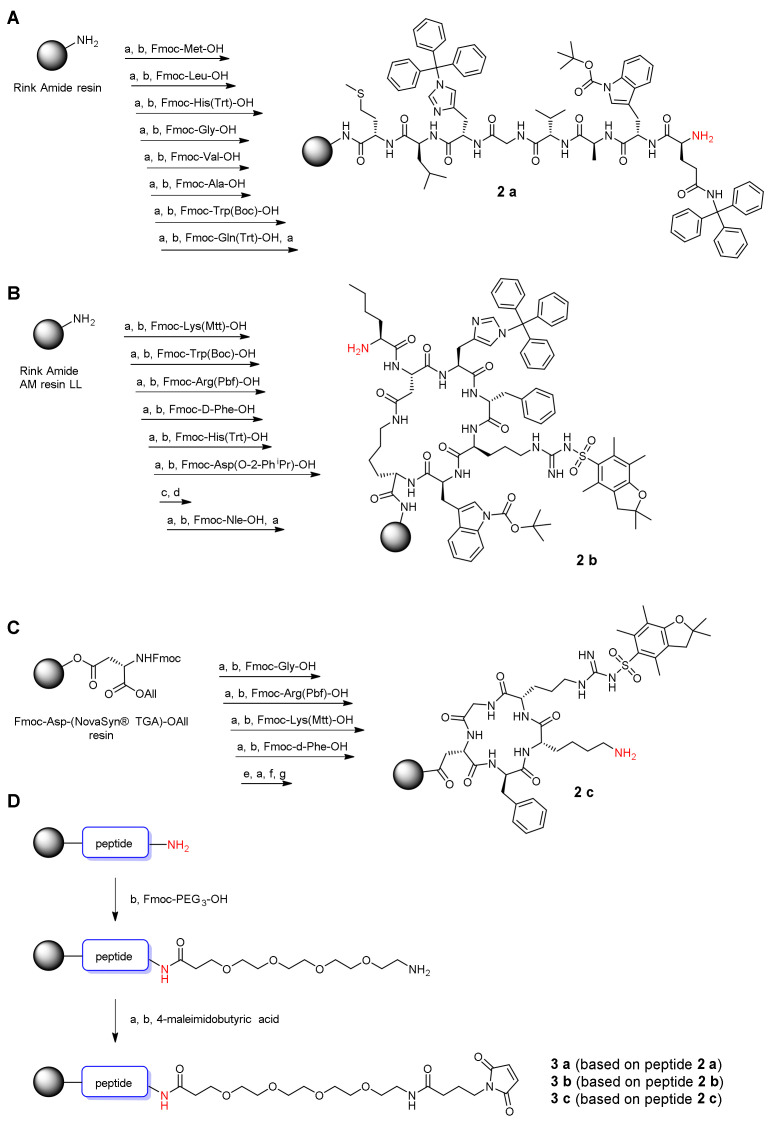
Solid-phase synthesis of peptide units **3 a-c**: (**A**) Synthesis of resin-bound protected BBN_7-14_ **2 a** (A), Nle-c(DHfRWK) **2 b** (**B**) and c(RGDfK) **2 c** (**C**). The amino function which was further functionalized with the linker is marked in red. (**D**) Peptide functionalization for the preparation of maleimide-modified peptide monomers **3 a-c**. Reaction conditions: (**a**) deprotection of Fmoc-PG: piperidine/DMF (1/1, *v/v*), 1 + 5 min; (**b**) activation of amino acids: 4.0 equiv. DIPEA, 3.9 equiv. HBTU in DMF, 2 min, coupling, 60 min; (**c**) deprotection of Mtt- and O-2-Ph^i^Pr-PG: TFA/CH_2_Cl_2_ (1/99, *v/v*), 90 min; (**d**) cyclization: 4.0 equiv. DIPEA, 3.9 equiv. PyBOP in DMF, 20 h; (**e**) deprotection of All-PG: 24.0 equiv. PhSiH_3_, 0.25 equiv. Pd(PPh_3_)_4_ in CH_2_Cl_2_, 3 × 30 min; (**f**) cyclization: 4.0 equiv. DIPEA, 3.9 equiv. HBTU in DMF, 16 h; (**g**) deprotection of Mtt-PG: TFA/CH_2_Cl_2_ (1/99, *v/v*), 3 × 30 min.

For this purpose, the peptides were synthesized on solid support (Scheme 1A-C) and in the following further modified using a PEG_3_ linker unit followed by 4-maleimidobutyric acid to obtain peptides analogs amenable to efficient chemoselective functionalization with the MIUs (Scheme 1D).

In detail, the three peptides **2 a-c** were completely built on solid support using standard Fmoc-based solid phase-assisted synthesis protocols, as reported in previous procedures [27,28,29]. Cyclization of peptides was performed while being still attached to the resin and after removal of the respective protecting groups (Mtt- and O-2-Ph^i^Pr-PG (**2 b**) were removed under mildly acidic conditions, and the All-PG (**2 c**) by treatment with phenylsilane (PhSiH_3_) and tetrakis(triphenylphosphine) palladium(0) (Pd(PPH_3_)_4_)). Following the final Fmoc-deprotection of **2 a-b** and the Mtt-deprotection of **2 c**, the maleimide-modified peptides **3 a-c** were prepared by introducing a linker composed of a polyethylene glycol spacer (PEG_3_) and 4-maleimidobutyric acid. The PEG_3_ linker was chosen as it was shown to give GRPR-specific BBN_7-14_-based agents with optimal properties (such as high tumor uptake and in vivo stability [30]) and was thus used for all agents for direct comparability of the obtained results. The maleimide function on the other hand allows to introduce the MIUs into the peptide structure via an efficient and chemoselective thiol-based Michael addition click reaction.

### 2.2. Synthesis of the Hybrid Multimodal Imaging Units **7**-**10**

**Scheme 2:**
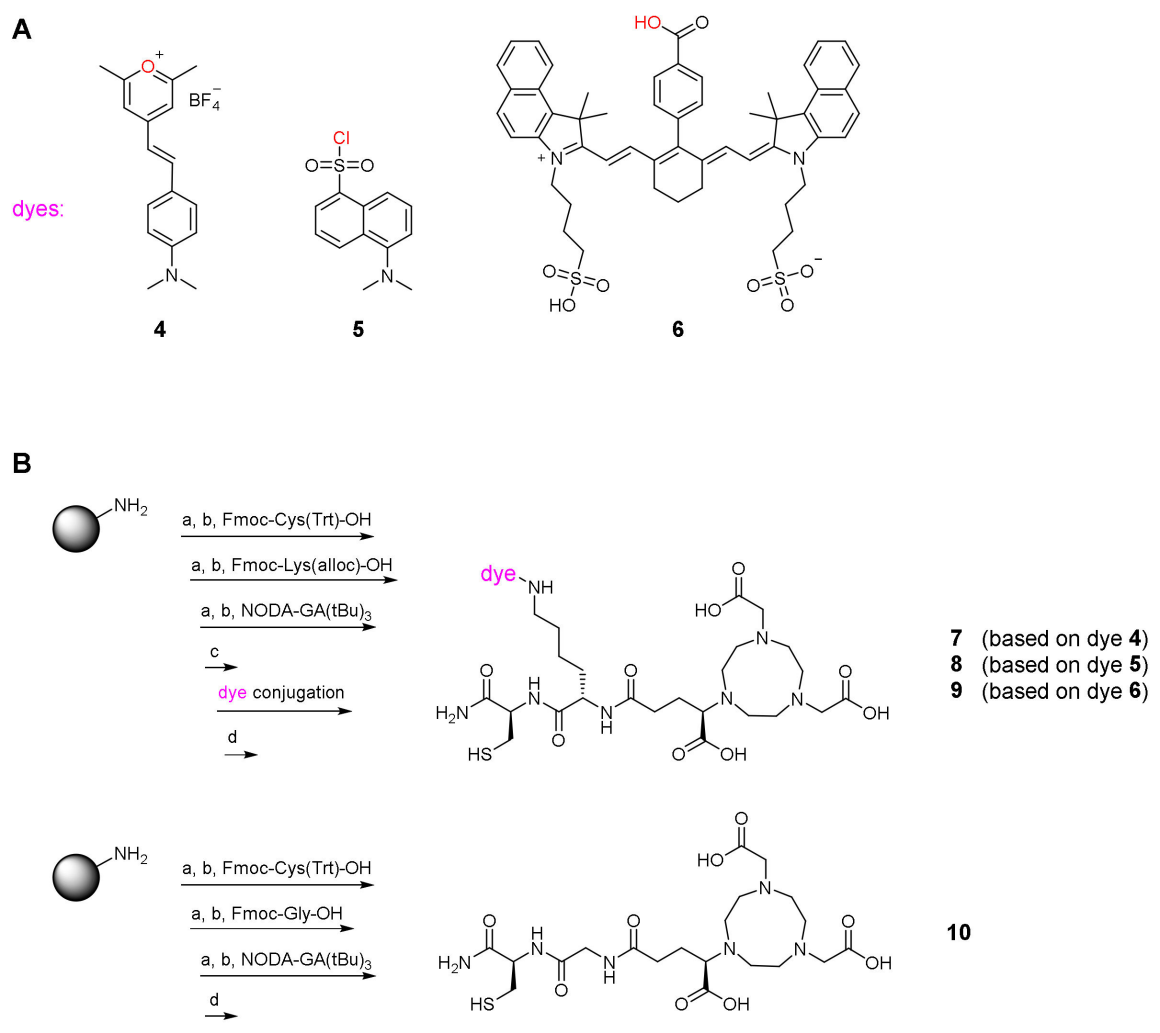
Structures of the fluorescent dyes **4-6** which were selected for the preparation of the MIUs **7-10** (**A**). The functional group of the respective dye which was reacted with the N_ε_-amine of the lysine in Cys(Trt)-Lys-NODA-GA(tBu)_3_ to obtain the MIUs is marked red. Solid phase-based synthesis of the MIUs **7-10** (**B**). Reaction conditions: (a) deprotection of Fmoc-PG: piperidine/DMF (1/1, *v/v*), 1 + 5 min; (b) activation of amino acids: 4.0 equiv. DIPEA, 3.9 equiv. HBTU in DMF, 2 min, coupling, 60 min; (c) deprotection of the alloc-PG: 20.0 equiv. morpholine, 2.0 equiv. Pd(PPh_3_)_4_ in CH_2_Cl_2_, 2 h; (d) cleavage from the resin and deprotection of the remaining protective groups: TFA/TIS (95/5, *v/v*), 1 h.

The molecular design of the MIUs was based on the structure of the previously reported PESIN-homodimer conjugates, in which the imaging synthons were composed of (1,4,7-triazacyclononane-4,7-diyl)diacetic acid-1-glutaric acid (NODA-GA) as chelating agent for ^68^Ga- or ^64^Cu-labeling, a variety of fluorescent small dye molecules for the optical detection and a thiol functional group for the efficient conjugation to the peptide vector [20,21]. In the present work, a pyrylium dye (Scheme 2A, **4**) [31], dansyl chloride (Scheme 2A, **5**) and a cyanine dye based on the structure of indocyanine green (Scheme 2A, **6**) [32] were chosen for their biological and optical properties, as well as for their different ionic charge, whose influence on the conjugated peptide moieties should be studied. The synthesis of the MIUs **7**-**9** based on these dyes was performed completely on solid support by employing an orthogonal protecting group strategy, as reported in Scheme 2B. Firstly, the NODA-GA-modified di-peptide Cys(Trt)-Lys(alloc)-NODA-GA(tBu)_3_ was prepared, followed by the deprotection of the N_ε_-alloc protecting group of lysine by palladium catalysis. The free N_ε_-amino group of lysine was then reacted with the corresponding dyes: the pyrylium dye **4** and dansyl chloride **5** were reacted directly necessitating no prior activation, while the cyanine dye **6** (prepared following a reported procedure) [27] was activated beforehand with HBTU and DIPEA. After cleavage from solid support and deprotection, the MIUs **7**-**9** (whose total charges—considering also the chelator—are −2, −3 and −4, respectively) were obtained in high purity and reasonable yields. Finally, it was necessary to synthesize an appropriate reference compound to compare the influence of the MIUs on the chemical, biological and photophysical properties of the resulting conjugates. For this purpose, the synthon **10** (net charge = −3), bearing only the NODA-GA chelator but lacking the fluorescent dye, was prepared (Scheme 2B).

### 2.3. Synthesis of the MIU-Peptide-Bioconjugates **7 a**-**c**, **8 a-c**, **9 a-c** and **10 a-c**

**Scheme 3:**
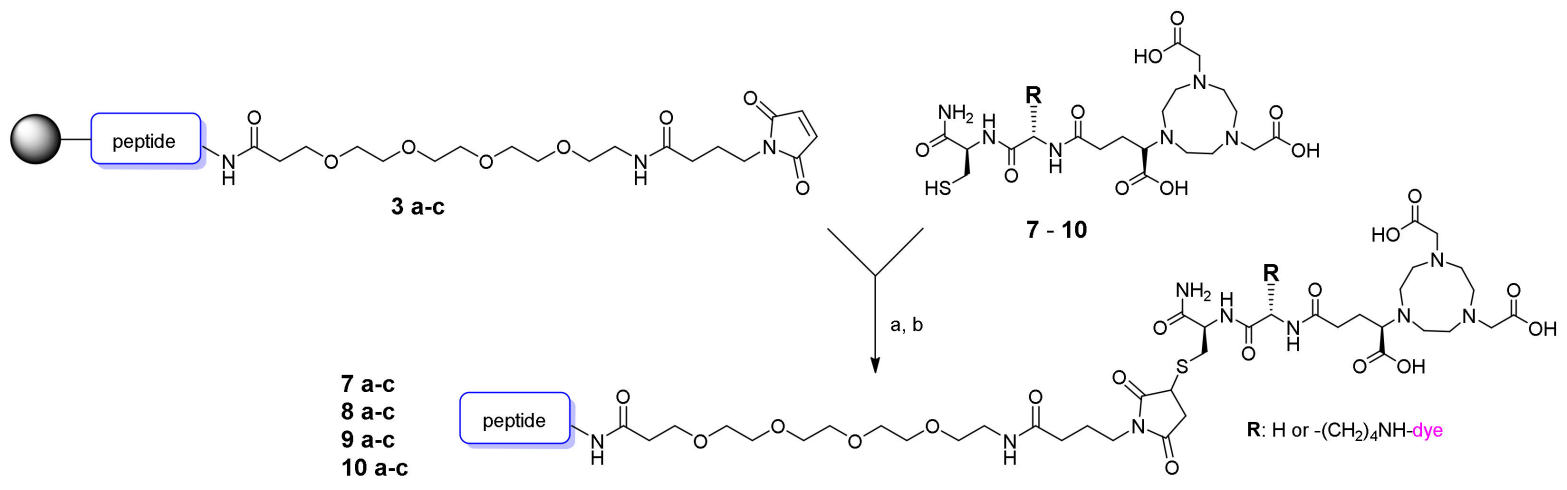
Schematic depiction of the conjugation reaction of the maleimide-modified peptides **3 a-c** with the corresponding MIUs **7**-**10** to the hybrid peptide-MIU-conjugates **7 a-c**, **8 a-c**, **9 a-c** and **10 a-c**. Reaction conditions: (a) conjugation of the building blocks: H_2_O/CH_3_CN (1/1, *v/v*), 5 min, pH = 7; (b) cleavage from resin: TFA/TIS (95/5, *v/v*), 1 h.

To assemble the hybrid multimodal imaging agents **7 a**-**c**, **8 a-c**, **9 a-c** and **10 a-c**, the MIUs **7**-**10** were reacted with the corresponding peptide units **3 a-c**. The reactions were carried out under mild conditions via the bioorthogonal Michael addition click reaction [33], resulting in the formation of the final conjugates in only 5 min (Scheme 3).

The non-optimized reaction yields obtained after the cleavage from the resin and the purification of the conjugates are given in Table 1 showing that the designed MIUs can be chemoselectively introduced in maleimide-comprising biomolecules with high efficiency, yielding the modified multimodal imaging agents in acceptable yields. Moreover, this modular approach enables both the tailored design of hybrid multimodal imaging units with different imaging properties by simply exchanging their used molecular building blocks (e.g., chelator derivative or fluorescent dye), as well as the efficient introduction of these units into the structure of varying peptidic vectors targeting different receptors and thus tumors.

### 2.4. Determination of Photophysical Properties of the MIU-Peptide-Bioconjugates **7 a-c, 8 a-c** and **9 a-c**

Following the synthesis of the target compounds, their photophysical properties were determined. Here, a possible negative influence of the conjugation of the different MIUs to the corresponding peptidic structures on the absorbance coefficients, or the absorption or emission wavelengths should be ruled out. In Table 2, the results of these evaluations are reported for the MIU-peptide-bioconjugates **7 a-c**, **8 a-c** and **9 a-c** as well as for the MIUs **7**-**9**. As expected, no significant loss or shift of fluorescence properties of the final compounds compared to their imaging units before conjugation was observed. This is in line with the earlier observation for PESIN homodimer-MIU-conjugates where an influence of dye or MIU bioconjugation on fluorescence quantum yields and other photophysical properties could be ruled out [20,21].

### 2.5. Radiolabeling of the MIU-Peptide-Bioconjugates **7 a-c**, **8 a-c**, **9 a-c** and **10 a-c** and Determination of the Lipophilicity/Hydrophilicity of the Labeled Agents

**Figure 3:**
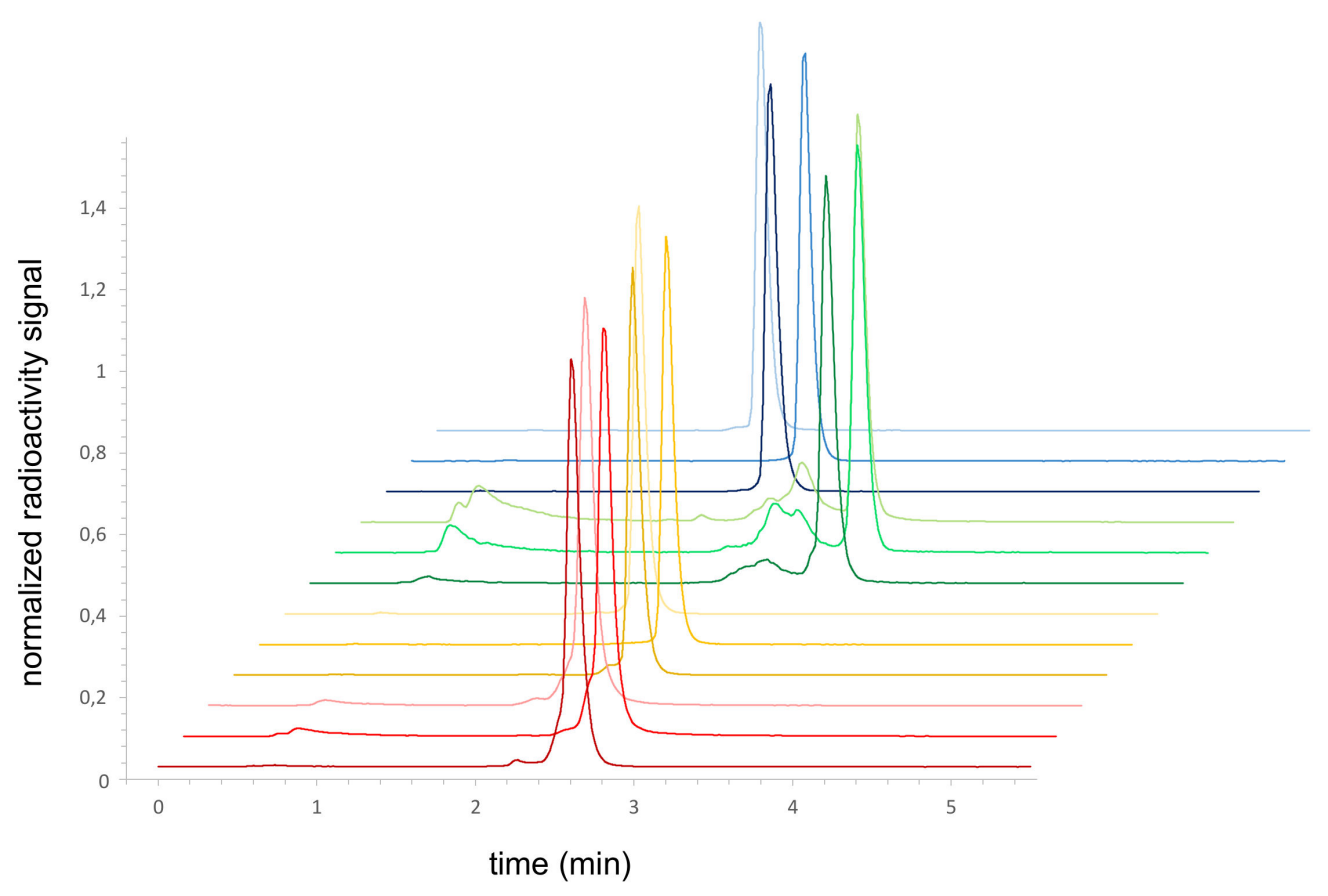
Radio-HPLC chromatograms of the synthesized imaging agents, from front to back: [^68^Ga]Ga-**7 a**, [^68^Ga]Ga-**7 b**, [^68^Ga]Ga-**7 c**, [^68^Ga]Ga-**8 a**, [^68^Ga]Ga-**8 b**, [^68^Ga]Ga-**8 c**, [^68^Ga]Ga-**9 a**, [^68^Ga]Ga-**9 b**, [^68^Ga]Ga-**9 c**, [^68^Ga]Ga-**10 a**, [^68^Ga]Ga-**10 b**, [^68^Ga]Ga-**10 c**.

The radiolabeling efficiency of the synthesized conjugates with ^68^Ga^3+^ (t_½_ = 68 min) was investigated in order to verify their radiochemical suitability for combined hybrid multimodal imaging using PET and OI. Radiolabeling of all the compounds with ^68^Ga was performed in aqueous solution at 45 °C for 10 min using standard acetate buffer conditions at pH 3.9–4.0 [34]. The addition of ascorbic acid to the reaction mixture showed to be mandatory in order to avoid the relatively fast degradation of the fluorescent dyes that were susceptible to the radiolysis-induced formation of ions and radicals within minutes [35]. Under these conditions, the labeled bioconjugates [^68^Ga]Ga-**7 a-c**, [^68^Ga]Ga-**8 a-c**, [^68^Ga]Ga-**9 a-c** and [^68^Ga]Ga-**10 a-c** were obtained in high radiochemical yields (RCYs) of ≥95% and high non-optimized molar activities of 90–120 GBq/µmol. Most of the compounds exhibited high radiochemical purities (RCPs) of ≥95%, except from [^68^Ga]Ga-**9 a-c** (carrying the indocyanine-based dye) which were obtained in lower RCPs of ≥85% (Figure 3). As this effect was not observed for the earlier-reported PESIN homodimer and tetramer of MIU **9** [20,21] and occurs in case of all three peptide conjugates, it is thus no result of dye- or peptide-related decomposition, but might be caused by the formation of aggregates of the peptide monomer conjugates due to the large size of the π-system of the dye as peptide monomers should be less able to hinder this aggregate formation compared to peptide multimers.

In the following, the lipophilicity of the labeled conjugates was investigated to get a first idea of the potential excretion pathway of the agents during an in vivo application. The log*_D_* values were determined by partition experiments between n-octanol and aqueous solution at pH 7.4. By comparing the obtained data with the results for the MIUs **7-10**, only a minor reduction in hydrophilicity was observed being caused by the introduction of the peptide monomers (Table 3). Compounds [^68^Ga]Ga-**10 a-b**, which lack the dye in their imaging synthon, presented the lowest lipophilicity of the tested compounds, with log_D_ values of −3.78 ± 0.04, −3.36 ± 0.02 and −4.06 ± 0.10, respectively.

**Figure 4:**
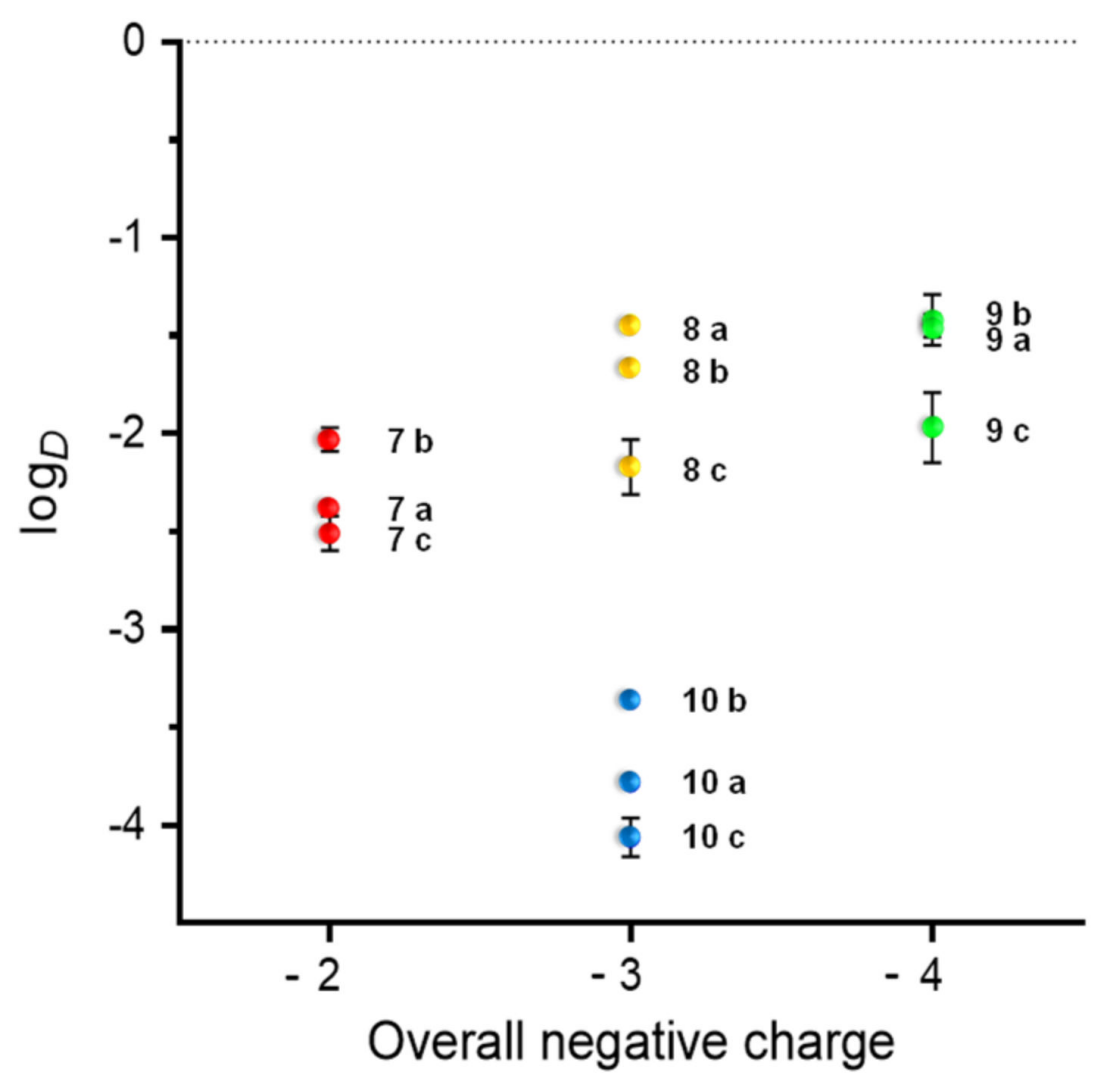
Plot of log*_D_* vs. overall negative charge of the radiolabeled MIU-peptide-bioconjugates [^68^Ga]Ga-**7 a-c**, [^68^Ga]Ga-**8 a-c**, [^68^Ga]Ga-**9 a-c** and [^68^Ga]Ga-**10 a-c**.

In general, the log*_D_*s of the bioconjugates seem to be mainly dependent on the structural composition of the hybrid imaging units, especially on their charge and the size of their π-conjugated systems (Figure 4). It can be deduced that the major factor influencing the compounds’ hydrophilicity/lipophilicity is the applied MIU, and within these systems, the main influence is exerted by the selected fluorescent dye.

### 2.6. Receptor-Specific Cell Staining and Fluorescence Microscopy

**Figure 5:**
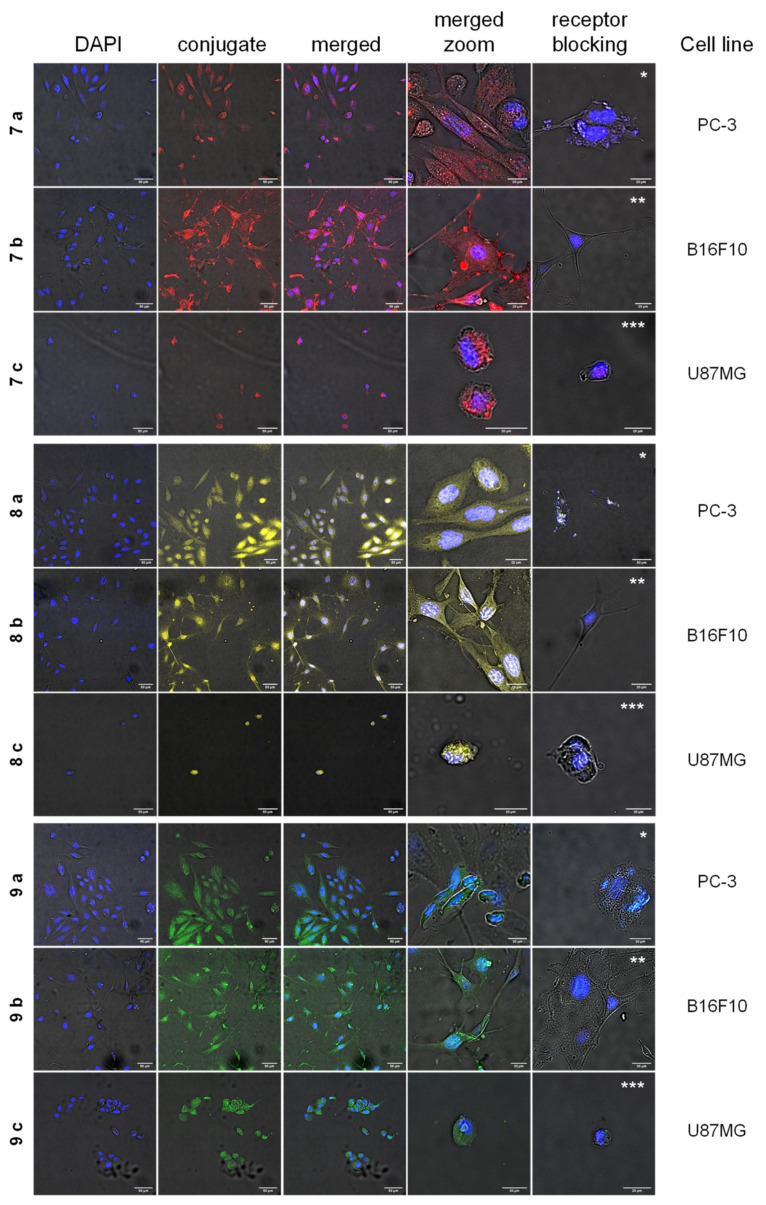
Receptor-specific staining of GRPR-positive PC-3, MC1R-positive B16F10 and integrin α_v_β_3_-positive U87MG tumor cells using 10 mM solutions of the hybrid multimodal bioconjugates **7 a-c** (red signal, detector window 705–751 nm), **8 a-c** (yellow signal, detector window 705–800 nm) and **9 a-c** (green signal, detector window 705–800 nm). The cell nuclei were stained with DAPI (detector window 440–490 nm). Receptor blocking experiments were performed applying a 50-fold excess of * BBN, ** NDP-MSH and *** c(RGDyK), respectively.

As all bioconjugates (**7a-9a**, **7b-9b** and **7c-9c**) showed favorable and preserved fluorescence properties, the agents were studied in terms of their ability to specifically visualize their respective target receptors (GRPR, MC1R or integrin α_v_β_3_) on tumor cells. For this purpose, the respective receptor-positive PC-3, B16F10 and U87MG cells, expressing GRPR, MC1R and integrin α_v_β_3_ receptors, respectively, were incubated with the corresponding hybrid multimodal agent and analyzed by confocal microscopy experiments. Receptor-mediated binding was confirmed for all of the compounds by detection of fluorescence at the expected wavelength (Figure 5). Nonspecific binding was excluded by receptor blocking experiments, using a 50-fold excess of appropriate blocking agent (endogenous bombesin for GRPR blocking, NDP-MSH for MC1R blocking and c(RGDyK) for integrin α_v_β_3_ blocking), reducing the membrane-associated fluorescence to background levels, thus demonstrating the specific accumulation of the multimodal imaging agents in the cells.

### 2.7. Determination of the In Vitro Receptor Binding Affinities of the Hybrid Peptide-MIU-Bioconjugates

**Figure 6:**
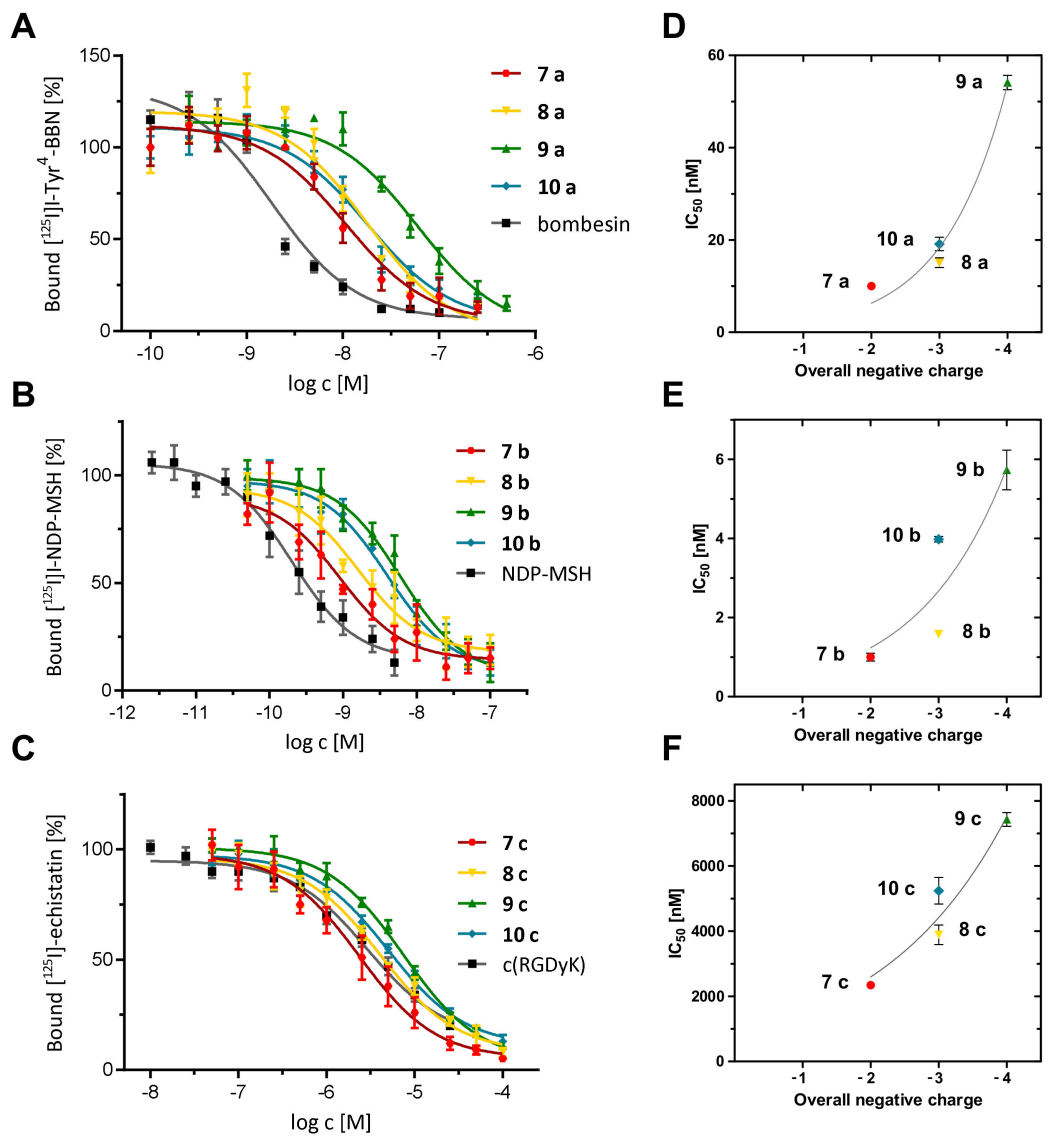
Results of the competitive displacement assays of the peptide-MIU-bioconjugates on different tumor cell lines. Depiction of the determined binding curves of: (**A**) **7 a**-**10 a** and the reference bombesin, tested on stably GRPR-transfected HEK-293 cells, using [^125^I]I-Tyr^4^-bombesin as the competitor, (**B**) **7 b**-**10 b** and the reference NDP-MSH, tested on MC1R-positive B16F10 cells, using [^125^I]I-NDP-MSH as the competitor, (**C**) **7 c**-**10 c** and the reference c(RGDyK), tested on α_v_β_3_-expressing U87MG cells, using [^125^I]I-echistatin as the competitor. Correlation between the found IC_50_ values and number of negative charges of: (**D**) **7 a**-**10 a**, (**E**) **7 b**-**10 b** and (**F**) **7 c**-**10 c**. Error bars represent SD.

The aim of the present study was directed to explore different aspects of the charge-receptor binding affinity-correlation previously described and therefore, competitive displacement assays were performed for the hybrid multimodal agents. The first question that was addressed was whether the introduction of the MIUs into PESIN monomers would result in the same effects than observed for the binding properties of the homodimer equivalents. Thus, conjugates **7 a**-**10 a**, which exhibit BBN_7-14_ monomer (**1 a**) as functional peptide, were tested on stably GRPR-transfected HEK-293 cells, using [^125^I]I-Tyr^4^-bombesin as the competitor and endogenous bombesin (BBN) as an internal standard with a known high affinity for this receptor type [27]. All the conjugates of this first series showed IC_50_ values in the range of 10.01 ± 0.67 nM to 58.27 ± 3.66 nM (Table 3, Figure 6A,D), while endogenous bombesin (BBN) exhibited an IC_50_ of 2.01 ± 0.17 nM. By comparing these data with the reported results of the corresponding homodimeric analogues reported before [20,21], it can be deduced that: (i) for PESIN monomers, the conjugation with the MIUs resulted in a decreased GRPR binding affinity compared to the endogenous reference BBN, confirming the results of the dimeric agents; (ii) the compound carrying the uncharged dansyl dye (**8 a**, overall net charge = −3) and the one lacking the fluorescent dye (**10 a**, overall net charge = −3) presented similar, slightly increased IC_50_ values (15.09 ± 1.04 nM and 19.13 ± 1.44 nM, respectively) compared to the conjugate containing the positively charged pyridinium dye (**7 a**, 10.01 ± 0.67 nM, overall net charge = −2); (iii) the cyanine-based conjugate **9 a**, carrying a negative charge within the fluorescent dye giving an overall net charge of −4, presented a considerably decreased GRPR affinity (IC_50_ value of 58.27 ± 3.66 nM), also being in accordance with the results obtained for the dimeric bioconjugates; (iv) compared to their homodimeric analogs, **7 a**, **8 a** and **10 a** showed a slight increment in the binding affinities (IC_50_ values of the corresponding homodimers were 16.47 ± 0.25 nM, 26.20 ± 0.89 nM and 21.48 ± 1.22 nM, respectively), whereas **9 a** more than doubled its IC_50_ value compared to the corresponding homodimer (exhibiting an IC_50_ of 27.39 ± 2.01 nM), thus significantly decreasing the affinity to the receptor.

Taken together, these data demonstrate that the conjugation of the MIUs to the monomeric PESIN peptide results in principle in only a minor reduction of binding affinity of the conjugates, which were still able to specifically interact with the GRPR with high affinity and the negative influence of anionically charged fluorescent dyes on GRPR binding affinities was confirmed. As expected, the adverse influence of the negatively charged dye on GRPR affinities was more pronounced in case of the peptide monomer compared to the peptide homodimer, confirming the theory that a higher number of peptide copies within the bioconjugates is at least in part able to compensate the adverse effect of the negative charge of the dye [22].

In the following, bioconjugates **7 b**-**10 b**, comprising Nle-c(DHfRWK) (**1 b**) as receptor-specific peptide, were tested on MC1R-expressing B16F10 melanoma cells, using [^125^I]I-NDP-MSH as the competitor and NDP-MSH as internal standard with known high affinity for this receptor type [36,37]. The compounds of this series showed IC_50_ values in the range of 1.00 ± 0.10 nM to 5.73 ± 0.50 nM (Table 3, Figure 6B,E), while the reference compound exhibited an IC_50_ value of 0.25 ± 0.02 nM. From the obtained data it becomes clear that: (i) the conjugate containing the uncharged dansyl dye (**8 b**, IC_50_ = 1.58 ± 0.04 nM, overall net charge = −3) presented again a similar IC_50_ value compared to the compound carrying the positively charged pyridinium dye (**7 b**, IC_50_ = 1.00 ± 0.10 nM, overall net charge = −2); and (ii) again, the lowest receptor affinity was found for the conjugate bearing the negatively charged cyanine-based conjugate **9 b** (IC_50_ = 5.73 ± 0.50 nM, overall net charge = −4). Taken together, these results show the same clear trend of negative correlation between anionically charged fluorescent dye and receptor affinity of the hybrid agent: The higher the overall negative charge of the conjugates was, the lower the resulting MC1R binding affinity.

Finally, conjugates **7 c**-**10 c**, comprising c(RGDfK) (**1 c**) as receptor-specific peptide, were tested on integrin α_v_β_3_-expressing U87MG tumor cells for their α_v_β_3_-affinity, using [^125^I]I-echistatin as the competitor and c(RGDyK) as internal standard with reasonable affinity for this receptor type [38]. The compounds of this final series showed IC_50_ values in the range of 2338.00 ± 116.83 nM to 7425.75 ± 207.44 nM (Table 3, Figure 6C,F), while the reference compound exhibited an IC_50_ value of 2936.60 ± 208.22 nM, being in accordance with literature data. From the assay results it can be deduced that: (i) compound **7 c**, carrying the positively charged pyridinium dye (overall net charge = −2), presented the highest α_v_β_3_ binding affinity (IC_50_ = 2338.00 ± 116.83 nM), being in accordance to the results obtained for the other receptor systems; (ii) the compound carrying the uncharged dye (**8 c**) and the one lacking the fluorescent dye (**10 c**), both characterized by an overall net charge of −3, presented similar IC_50_ values (3893.50 ± 295.89 nM and 4560.67 ± 436.66 nM, respectively), being also in accordance with the results found for the other receptor-specific agents discussed before; and (iii) again, the compound carrying the negatively charged dye (**9 c**, overall net charge = −4) presented the lowest α_v_β_3_ binding affinity (IC_50_ = 7425.75 ± 207.44 nM), also confirming the results discussed before.

## 3. Materials and Methods

### 3.1. Materials and Methods

All commercially available chemicals and solvents were at least of analytical grade and used, if not otherwise stated, without further purification. Fmoc-protected amino acids, Benzotriazole-1-yl-oxy-tris-pyrrolidino-phosphonium hexafluorophosphate (PyBOP), and the resins for the peptide synthesis (Rink Amide resin, Rink Amide AM resin LL, Fmoc-Asp-(NovaSyn^®^ TGA)-OAll resin with loading 0.54, 0.34 and 0,18 mmol/g, respectively) were purchased form NovaBiochem (Darmstadt, Germany). 15-(9-Fluorenylmethyloxycarbonyl)amino-4,7,10,13-tetraoxa-pentadecanoic acid (PEG_3_, Fmoc-NH-PEG_3_-COOH) was obtained from Iris Biotech (Marktredwirz, Germany), 4-maleimidobutyric acid from ABCR (Karlsruhe, Germany), and 4-(4,7-*bis*(2-(t-butoxy)-2-oxoethyl)-1,4,7-triazacyclononan-1-yl)-5-(*tert*-butoxy)-5-oxopentanoic acid ((*R*)-NODA-GA(*t*Bu)_3_) from CheMatech (Dijon, France). 2,4,6-trimethylpyrylium tetrafluoroborate, 4-dimethylaminobenzaldehyde, 4-carboxyphenylboronic acid and *tetrakis*(triphenylphosphine)palladium(0) (Pd(PPh_3_)_4_) were purchased from TCI (Eschborn, Germany), morpholine, *N*,*N*-diisopropylethylamine (DIPEA), triisopropylsilane (TIS), 5-(dimethylamino)naphthalene-1-sulfonyl chloride (Dansyl-Chloride) and IR820 from Sigma-Aldrich (Taufkirchen, Germany). Dichloromethane (DCM), diethylether, dimethylformamide (DMF), 2-(1H-benzotriazol-1-yl)-1,1,3,3-tetramethyluronium hexafluoro-phosphate (HBTU), trifluoroacetic acid (TFA) and water were purchased from Carl Roth (Karlsruhe, Germany), acetonitrile (MeCN) from Häberle Labortechnik (Lonsee-Ettlenschieß, Germany).

For HPLC chromatography, a Dionex UltiMate 3000 system was used together with Chromeleon Software (Version 6.80). For semipreparative analyses, a Chromolith (RP-18e, 100–10 mm, Merck, Germany) column was used. For radioanalytical use, a Dionex UltiMate 3000 system equipped with a Raytest GABI Star radioactivity detector was used together with a Chromolith Performance (RP-18e, 100–4.6 mm, Merck, Germany) column. All operations were performed with a flow rate of 4 mL/min using H_2_O + 0.1% TFA and MeCN + 0.1% TFA as solvents. HR-ESI (high-resolution Electrospray Ionization) and MALDI (Matrix-Assisted Laser Desorption/Ionization) spectra were obtained with Finnigan MAT95Q and Bruker Daltronics Microflex spectrometers, respectively. γ-counting was performed using a 2480 Wizard gamma counter system from Perkin Elmer (Rodgau, Germany). For absorbance and emission measurements, a Tecan Infinite M200 Microplate reader together with a Nunc Micro-Well 96 solid plate from ThermoFisher (Dreieich, Germany) was used. Confocal fluorescence microscopy was performed on a Leica TCS SP8 confocal microscope with lasers at λ = 405, 488, 552 and 638 nm. Overlays of microscopies were generated directly with the operating software or afterwards using FIJI software (V1.50e).

**7 a**-**10 a** conjugates were tested on Transfected Human Embryonic Kidney 293 cells stably expressing the GRP-Receptor (HEK-GRPR). HEK-GRPR cells were obtained from Dr. Martin Béhé, Paul Scherrer Institute, Villingen, Switzerland. **7 b**-**10 b** and **7 c**-**10 c** conjugates were tested on Murine Melanoma cells (B16F10) and Human Glioblastoma cells (U87MG), respectively, which were purchased from ATCC (Wesel, Germany). Confocal microscopy of BBN_7-14_ conjugates was performed using the human tumor cell line PC-3 (GRPR-positive), which was obtained from DSMZ (Braunschweig, Germany). [^125^I]-Tyr^4^-bombesin, [^125^I]-NDP-MSH and [^125^I]-echistatin were purchased from Perkin Elmer (Rodgau, Germany) in a molar activity of 81.4 GBq/µmol. Dulbecco’s Modified Eagle’s Medium (DMEM, high glucose, GlutaMax-I, 500 mL), Dulbecco’s Modified Eagle’s Medium (DMEM, 1×, 500 mL), geneticin (G418 Sulfate, 50 mg/mL), Opti-MEM I (GlutaMAX I), RPMI 1640 medium, L-Glutamine and PenStrep were obtained from Gibco (Schwerte, Germany), Eagle’s Minimum Essential Medium (EMEM, 500 mL) from ATCC (Wesel, Germany), FCS (fetal calf serum) from Bio&SELL (Feucht, Germany) and Dulbecco’s phosphate buffered saline (PBS), 1,10-phenantroline, *tris*(hydroxymethyl)aminomethane hydrochloride (Tris·HCl), manganese chloride (MnCl_2_), 0.25% Trypsin with 0.02% EDTA solution in PBS and 4-(2-Hydroxyethyl)piperazine-1-ethanesulfonic acid (HEPES) from Sigma-Aldrich (Taufkirchen, Germany). Bovine serum albumin (BSA), sodium chloride (NaCl), calcium chloride (CaCl_2_) and magnesium chloride (MgCl_2_) were purchased from CarlRoth (Karlsruhe, Germany). The ^68^Ge/^68^Ga-Generator used was an IGG100 system, obtained from Eckert & Ziegler (Berlin, Germany) and eluted with HCl (0.1 M, 1.6 mL).

Analytical details and original spectra of the new compounds are available in the Supplementary Materials (Figures S1–S16).

### 3.2. General Synthesis of Peptides

Peptides were synthesized on Rink Amide resins by using Nα-Fmoc protecting groups and a standard HBTU activation strategy [39]. The resin was swollen in DCM for 30 min, washed with DMF, the Fmoc protecting group was cleaved with piperidine (50% in DMF, 2 min washing then 5 min). The resin was washed with DMF, then the respective protected amino acid was coupled by using the HBTU-pre-activated synthon in DMF (4 equiv. Nα-Fmoc amino acid, 3.9 equiv. HBTU, 4 equiv. DIPEA) which was allowed to react for 2 min before being added to the resin. The syringe was shaken for 1 h, then the reaction mixture was removed and the resin was washed with DMF. The same procedure was repeated for the following amino acids. Detailed syntheses of the three peptide units can be found in the corresponding references [27,28,29]. The PEG spacer (4 equiv.) as well as 4-maleimido butyric acid (4 equiv.) were coupled following the procedure previously described [39]. Then the resin was washed thrice with DMF, dichloromethane and diethyl ether, and dried under reduced pressure. Finally, the peptides were cleaved from solid support by using a mixture of TFA:TIS (95:5 (*v/v*), 5 mL) for 1 h. The volatile components were removed under reduced pressure, the residues were dissolved in 1:1 MeCN:H_2_O + 0.1% TFA and the products purified by semipreparative HPLC.

### 3.3. General Synthesis of the Multimodal Imaging Units (MIUs) **7**-**10**

Fluorescent dyes **4** and **6** were synthesized following previously reported procedures [20,21]. Rink amide resin-Cys(Trt)-Lys(alloc)-NODA-GA(*t*Bu)_3_ was synthesized according to the standard Fmoc-based solid phase peptide synthesis protocol reported earlier, then the allyloxycarbonyl protecting group was removed still on solid support using tetrakis(triphenylphosphine)palladium(0). In the following step, 50 µmol Rink amide resin-Cys(Trt)-Lys-NODA-GA(*t*Bu)_3_ was reacted with the respective dye **4**-**6**. MIUs **7** + **8**: 4 eq. of the dyes **4** or **5** were reacted with the resin for 1 h without prior activation, 4 eq. of DIPEA were added after 30 min. MIU **9**: dye **6** (1.5 eq.) was activated beforehand with HBTU (1.425 eq.) and DIPEA (1.5 eq.) as base, in DMF for 10 min, then reacted with the resin at 80 °C for 3 h. After the conjugation reaction was finished, the resin was filtered from the liquid components of the mixture and washed thrice with DMF, dichloromethane and diethyl ether. After drying, the dye conjugates were cleaved from solid support by using a mixture of TFA:TIS (95:5 (*v/v*), 5 mL) for 1–2 h. Then the volatile components were removed under reduced pressure, the residues were dissolved in 1:1 MeCN:H_2_O + 0.1% TFA and the products purified by semipreparative HPLC. Similarly, Rink amide resin-Cys(Trt)-Gly-NODA-GA(*t*Bu)_3_ **10** was prepared following the standard Fmoc-based solid phase peptide synthesis protocol, washed thrice with DMF, dichloromethane and diethyl ether, and cleaved from solid support by using a mixture of TFA:TIS (95:5 (*v/v*), 5 mL) for 1 h. The dried residue was dissolved in 1:1 MeCN:H_2_O + 0.1% TFA and purified by semipreparative HPLC.

**7** (C_41_H_61_N_8_O_9_S)(CF_3_CO_2_): HPLC gradient: 0–100 % MeCN + 0.1% TFA in 12 min, R_t_ = 5.83 min, yield: 30%, purity: 98%, MALDI-MS (*m/z*) for [M]^+^ (calculated): 841.25 (841.43); HR-ESI-MS (*m/z*) for [M − 2H + K + Na]^+^ (calculated): 901.3257 (901.3655).

**8** (C_36_H_54_N_8_O_11_S_2_): HPLC gradient: 0–100% MeCN + 0.1% TFA in 12 min, R_t_ = 5.25 min, yield: 35%, purity: 85%, MALDI-MS (*m/z*) for [M + H]^+^ (calculated): 839.48 (839.34); [M+Na]^+^ (calculated): 861.49 (861.33); [M + K]^+^ (calculated): 877.50 (877.30); HR-ESI-MS (*m/z*) [M − H + K + Na]^+^ (calculated): 899.2405 (899.3353).

**9** (C_77_H_97_N_9_O_16_S_3_): HPLC gradient: 0–100 % MeCN + 0.1 % TFA in 5 min, R_t_ = 2.80 min, yield: 30 %, purity: 95 %, MALDI-MS (*m/z*) for [M + H]^+^ (calculated): 1500.49 (1500.62); [M + Na]^+^ (calculated): 1522.48 (1522.61); [M + K]^+^ (calculated): 1538.49 (1538.59); [M + 2Na]^+^ (calculated): 1545.38 (1545.60); HR-ESI-MS (*m/z*) for [M + 2K + Na]^2+^ (calculated): 779.7598 (779.8108).

**10** (C_20_H_34_N_6_O_9_S): HPLC gradient: 0–100% MeCN + 0.1% TFA in 8 min, R_t_ = 2.80 min, yield: 30%, purity: 95%, MALDI-MS (*m/z*) for [M + H]^+^ (calculated): 535.10 (535.59). HR-ESI-MS (*m/z*) for [M + H]^+^ (calculated): 535.2182 (535.5900).

### 3.4. General Synthesis of the Peptide-MIU-Conjugates **7 a-10 a**, **7 b-10 b** and **7 c-10 c**

1.45 µmol of the respective peptide (1 eq.) and 1.60 μmol of the respective MIU (1.1 eq.) were dissolved in 200 μL of 1:1 MeCN:H_2_O + 0.1% TFA and the pH was adjusted to 7.0 with phosphate buffer (0.5M, pH = 7.2). After 5 min of reaction at 25 °C, the HPLC purification of the products was performed.

**7 a** (C_103_H_153_N_23_O_26_S_2_): HPLC gradient: 0–100% MeCN + 0.1% TFA in 8 min, R_t_ = 6.02 min, yield: 76%, purity: 99%, MALDI-MS (*m/z*) for [M]^+^ (calculated): 2193.09 (2193.61); HR-ESI-MS (*m/z*) for [M + H]^2+^ (calculated): 1097.54822 (1097.305).

**8 a** (C_98_H_147_N_23_O_28_S_3_): HPLC gradient: 0–100% MeCN + 0.1% TFA in 12 min, R_t_ = 5.81 min, yield: 30%, purity: 98%, MALDI-MS (*m/z*) for [M + H]^+^ (calculated): 2190.27 (2190.57); HR-ESI-MS (*m/z*) for [M + 2H]^2+^ (calculated): 1096.5056 (1096.7850).

**9 a** (C_139_H_188_N_24_O_33_S_4_): HPLC gradient: 0–100% MeCN + 0.1% TFA in 5 min, R_t_ = 4.55 min, yield: 36%, purity: 99%, MALDI-MS (*m/z*) for [M + H]^+^ (calculated): 2851.70 (2851.42); HR-ESI-MS (*m/z*) for [M + Na]^2+^ (calculated): 1438.1405 (1438.2115).

**10 a** (C_82_H_125_N_21_O_26_S_2_): HPLC gradient: 0–100% MeCN + 0.1% TFA in 8 min, R_t_ = 4.52 min, yield: 54%, purity: 99%, MALDI-MS (*m/z*) for [M + H]^+^ (calculated): 1885.70 (1885.14); HR-ESI-MS (*m/z*) for [M + H]^2+^ (calculated): 943.9417 (943.8523).

**7 b** (C_108_H_155_N_25_O_25_S): HPLC gradient: 0–100% MeCN + 0.1% TFA in 5 min, R_t_ = 3.87 min, yield: 70%, purity: 99%, MALDI-MS (*m/z*) for [M + H]^+^ (calculated): 2234.38 (2234.65); HR-ESI-MS (*m/z*) for [M + 3H]^3+^ (calculated): 746.0528 (746.5534).

**8 b** (C_103_H_149_N_25_O_27_S_2_): HPLC gradient: 0–100% MeCN + 0.1% TFA in 5 min, R_t_ = 3.78 min, yield: 47%, purity: 98%, MALDI-MS (*m/z*) for [M + H]^+^ (calculated): 2232.15 (2232.59); HR-ESI-MS (*m/z*) for [M + 2H]^2+^ (calculated): 1117.5322 (1117.2954).

**9 b** (C_144_H_192_N_26_O_32_S_3_): HPLC gradient: 0–100% MeCN + 0.1% TFA in 5 min, R_t_ = 4.53 min, yield: 82%, purity: 99%, MALDI-MS (*m/z*) for [M + H]^+^ (calculated): 2895.18 (2895.45); HR-ESI-MS (*m/z*) for [M + H]^2+^ (calculated): 1448.1771 (1448.2250).

**10 b** (C_87_H_129_N_23_O_25_S): HPLC gradient: 0–75% MeCN + 0.1% TFA in 10 min, R_t_ = 5.96 min, yield: 52%, purity: 98%, MALDI-MS (*m/z*) for [M + H]^+^ (calculated): 1928.49 (1928.19); HR-ESI-MS (*m/z*) for [M + H]^2+^ (calculated): 965.4712 (965.0955).

**7 c** (C_87_H_129_N_19_O_24_S): HPLC gradient: 0–100% MeCN + 0.1% TFA in 8 min, R_t_ = 4.48 min, yield: 38%, purity: 99%, MALDI-MS (*m/z*) for [M + H]^+^ (calculated): 1854.76 (1855.17); HR-ESI-MS (*m/z*) for [M + 2H]^2+^ (calculated): 929.4675 (929.0854).

**8 c** (C_82_H_123_N_19_O_26_S_2_): HPLC gradient: 0–100% MeCN + 0.1% TFA in 5 min, R_t_ = 3.50 min, yield: 49%, purity: 99%, MALDI-MS (*m/z*) for [M + H]^+^ (calculated): 1853.62 (1853.11); HR-ESI-MS (*m/z*) for [M + Na + K]^2+^ (calculated): 958.8803 (958.5556).

**9 c** (C_123_H_166_N_20_O_31_S_3_): HPLC gradient: 0–100% MeCN + 0.1% TFA in 8 min, R_t_ = 5.80 min, yield: 33%, purity: 99%, MALDI-MS (*m/z*) for [M + H]^+^ (calculated): 2514.05 (2514.97); HR-ESI-MS (*m/z*) for [M + Na]^2+^ (calculated): 1270.0586 (1269.9852).

**10 c** (C_66_H_103_N_17_O_24_S): HPLC gradient: 0–100% MeCN + 0.1% TFA in 8 min, R_t_ = 3.96 min, yield: 23%, purity: 97%, MALDI-MS (*m/z*) for [M + H]^+^ (calculated): 1549.56 (1549.71); HR-ESI-MS (*m/z*) for [M + Na + K]^2+^ (calculated): 806.8173 (806.8556).

### 3.5. Radiochemistry

A solution of the peptide-MIU-conjugates in H_2_O (Tracepur quality, 1 mM) was added to 90–120 MBq of [^68^Ga]GaCl_3_ in a solution obtained by fractioned elution of an IGG ^68^Ge/^68^Ga generator system with HCl (0.1 M, 1.6 mL) and subsequent titration to pH 3.5–4.2 by addition of sodium acetate solution (1.25 M, 50–75 µL). All labeling experiments were performed by addition of 1 mg ascorbic acid to suppress radiolysis-induced product fragmentation. After 10 min of reaction at 45 °C, the mixtures were analyzed by analytical radio-HPLC. The radiolabeled products were found to be 95–99 % pure and obtained in non-optimized molar activities of 90–120 GBq/µmol.

### 3.6. Log_D_ Determination

The water/1-octanol partition coefficient (log*_D_*) was determined by adding 5 µL of the respectively ^68^Ga-labeled compound (0.8–1.2 MBq) to a mixture of phosphate buffer (0.05 M, pH 7.4, 795 µL) and 1-octanol (800 µL). The mixtures were intensively shaken for 5 min on a vibrating plate. After subsequent centrifugation at 13,000 rpm for 5 min, 125 µL were taken from each phase and measured in a γ-counter. The log*_D_* values were calculated from three or four independent experiments, each performed in triplicate.

### 3.7. Cell Culture

All cell lines were cultivated at 37 °C in a humidified incubator at 5% CO_2_. HEK-GRPR cells were cultured in Dulbecco’s Modified Eagle’s Medium (DMEM, high glucose, GlutaMax-I, 500 mL) supplemented with 10% FCS, 1.5% geneticin and 1% PenStrep. B16F10 cells were cultured in Dulbecco’s Modified Eagle’s Medium (DMEM, 1X, 500 mL) and the U87MG cells in Eagle’s Minimum Essential Medium (EMEM), each medium supplemented with 10% FCS and 1% PenStrep. The medium was exchanged every two or three days and cells were split at 70–90% confluence using 0.25/0.02% Trypsin/EDTA (*w*/*v*) in PBS. A medium change was performed 24 h before an experiment.

### 3.8. In Vitro Competitive Displacement Assays

To determine the binding affinity to the respective receptor, competitive displacement studies were performed on GRPR-expressing HEK, MC1R-expressing B16F10 and on integrin α_v_β_3_-expressing U87MG cells. Each compound was evaluated at least three times, each experiment being performed in triplicate. As radioligands, [^125^I]-Tyr^4^-bombesin, [^125^I]-NDP-MSH and [^125^I]-echistatin (81.4 GBq/μmol) were used as competitors. A Millipore Multiscreen punch kit and Millipore 96 well filter plates (pore size 1.2 µm) were used. The plates were incubated with PBS/BSA (1%) solution (each well 200 µL) at 25 °C for one hour before use. After preparing the respective binding buffers (Opti-MEM I-GlutaMAX I without additional supplements; DMEM with 25 mM HEPES, 0.3 mM 1,10-phenanthroline and 0.2% BSA; EMEM with 20 mM Tris·HCl, 150 mM NaCl, 2 mM CaCl_2_, 1 mM MgCl_2_, 1 mM MnCl_2_ and 0.1% BSA), the dilution series of the BBN_7-14_ (0.1–250 nM for **7 a**-**9 a**, 0.25–500 nM for **10 a**), Nle-c(DHfRWK) (0.005–100 nM for **7 b-10 b**), c(RGDfK) conjugates (50–100,000 nM for **7 c**-**10 c**) and the reference compounds (0.1–250 nM for endogenous BBN, 0.0025–5 nM for NDP-MSH, 10–25,000 nM for c(RGDyK)) were prepared in the respective binding buffer. The solution of the respective radioligand was prepared by adding 55–75 kBq of the respective ^125^I-labeled competitor to 7 mL of binding buffer. The respective cells were harvested and re-suspended in the binding buffer to give a cell concentration of 2 × 10^6^/mL. After the BSA solution was filtered using the Millipore Multiscreen vacuum manifold, 50 µL of a cell suspension containing 10^5^ cells were seeded in each well. Subsequently, 25 µL of the ^125^I-labeled competitor solution (0.01 kBq/µL) and 25 µL of the respective compound to be tested were added. The compound to be tested was added in eleven increasing concentrations, while the 12th well contained no test compound to ensure the 100% binding of the ^125^I-labeled competitor. After incubation of the plate for another hour at 25 °C, the solution was filtrated, and the cells were washed three times with cold PBS (1 × 200 µL, 2 × 100 µL). Using a Millipore MultiScreen disposable punch and a Millipore MultiScreen punch kit, the filters of the well plate were collected in γ-counter tubes separately and measured by γ-counting. The determination of the half-maximal inhibitory concentration (IC_50_) values was performed by fitting the obtained data via nonlinear regression using GraphPad Prism (v5.01).

### 3.9. Confocal Fluorescence Microscopy

Since the HEK-GRPR cells do not exhibit any adhered characteristics to glass plates, the GRPR positive human tumor cell line PC-3 was used for the confocal fluorescence microscopy experiments of the BBN_7-14_ series instead. PC-3 cells were cultured at 37 °C in RPMI 1640 medium supplemented with 10% FCS, 1% L-Glutamine and 1% PenStrep in a humidified atmosphere containing 5% CO_2_. B16F10 and U87MG cell lines were cultured as previously described for the competitive binding assays. Fluorescence microscopy was performed on a Leica TCS SP8 confocal microscope with laser excitation wavelengths of 405 and 638 nm. Overlays of microscopies were generated directly with the operating software or afterwards using FIJI software (v1.50e). Each cell line (4 × 10^4^) was seeded two days prior to the measurements in 24-well culture plates, each well was equipped with cover slips for microscopy. Medium was exchanged after 24h. After 2 days, the medium was removed and the cells were washed carefully with PBS. Then, 500 μL of a 10 μM solution of the peptide-MIU-conjugates in antibiotic-free medium were added, with or without the peptide blockade (BBN, NDP-MSH, c(RGDyK) 200 μM) followed by incubation for 4h. After washing of each cover slip with PBS thrice, they were consequently put reversed on object slides which were before prepared with a drop (10 μL) of DAPI antifade solution and then the respective cover slide was sealed and fixed with clear coat on the object slides.

## 4. Conclusions

All developed hybrid, dually labeled monovalent peptidic agents were found to receptor-specifically interact with the different target tumor cells. It was found that negatively charged fluorescent dyes resulted in a significant decrease in receptor binding affinities of the respective conjugates and this effect was found to be even more pronounced as in case of peptide homomultimers. Furthermore, this effect was not only found for GRPR-specific multimodal BBN_7-14_-based peptides, but also for the other peptide-receptor-systems studied and thus seems to be irrespective of the examined peptide-receptor system.

These results suggest that when designing peptidic multimodal compounds for use in PET/OI, the choice of the fluorescent dye to be used should be made very carefully, particularly in terms of charge, especially with regard to the receptor affinity of the conjugates to be obtained.

## Figures and Tables

**Table 1:** Non-optimized conjugation yields of the purified hybrid imaging agents **7 a-c**, **8 a-c**, **9 a-c** and **10 a-c** obtained by conjugation of the MIUs **7-10** to the peptide maleimides **3 a-c**.

Peptide Unit	MIU	Resulting Conjugate	Yield [%]
**3 a** (BBN_7-14_-maleimide)	**7** (thio-pyrylium-NODA-GA)	**7 a** (BBN_7-14_-pyrylium-NODA-GA)	76
	**8** (thio-dansyl-NODA-GA)	**8 a** (BBN_7-14_-dansyl-NODA-GA)	30
	**9** (thio-cyanine-NODA-GA)	**9 a** (BBN_7-14_-cyanine-NODA-GA)	36
	**10** (thio-NODA-GA)	**10 a** (BBN_7-14_-NODA-GA)	54
**3 b** (Nle-c(DHfRWK)-maleimide)	**7** (thio-pyrylium-NODA-GA)	**7 b** (Nle-c(DHfRWK)-pyrylium-NODA-GA)	70
	**8** (thio-dansyl-NODA-GA)	**8 b** (Nle-c(DHfRWK)-dansyl-NODA-GA)	47
	**9** (thio-cyanine-NODA-GA)	**9 b** (Nle-c(DHfRWK)-cyanine-NODA-GA)	82
	**10** (thio-NODA-GA)	**10 b** (Nle-c(DHfRWK)-NODA-GA)	52
**3 c** (c(RGDfK)-maleimide)	**7** (thio-pyrylium-NODA-GA)	**7 c** (c(RGDfK)-pyrylium-NODA-GA)	38
	**8** (thio-dansyl-NODA-GA)	**8 c** (c(RGDfK)-dansyl-NODA-GA)	49
	**9** (thio-cyanine-NODA-GA)	**9 c** (c(RGDfK)-cyanine-NODA-GA)	33
	**10** (thio-NODA-GA)	**10 c** (c(RGDfK)-NODA-GA)	23

**Table 2:** Summary of the photophysical properties of the MIUs **7-9** and the corresponding hybrid multimodal bioconjugates **7 a-c**, **8 a-c** and **9 a-c**. Values were determined in PBS at a concentration of 1 × 10^−5^ mol × L^−1^.)

Compound	λ_max(abs)_ (nm)	log ε (M^−1^cm^−1^)	λ_max(em)_^1^ (nm)	Stokes Shift (nm)
**7** ^3^	440	4.16	600	160
**7 a**	448	4.27	601	153
**7 b**	450	4.16	597	147
**7 c**	450	4.29	607	157
**8** ^3^	-	-	550	-
**8 a**	-	-	549	-
**8 b**	-	-	545	-
**8 c**	-	-	543	-
**9** ^2,3^	720/790	4.80/4.61	800	10
**9 a** ^2^	724/800	4.79/4.60	809	9
**9 b** ^2^	726/802	4.78/4.57	807	5
**9 c** ^2^	722/798	4.65/4.54	811	13

^1^ excitation wavelength λ_ex_ = 400 nm; ^2^ excitation wavelength λ_ex_ = 650 nm; ^3^ values of MIUs reported in literature [20,21].

**Table 3:** Summary of the log*_D_* values of the MIUs **7**-**9** and the corresponding hybrid bioconjugates [^68^Ga]Ga-**7 a-c**, [^68^Ga]Ga-**8 a-c**, [^68^Ga]Ga-**9 a-c** and [^68^Ga]Ga-**10 a-c** as well as of the receptor affinity data (IC_50_ values) measured on the respective cell lines. Values are given as mean ± SD.

Compound	log_D_	Overall Charge	IC_50_ (nM)
**7** ^4^	−2.04 ± 0.11	−2	-
**7 a**	−2.38 ± 0.02	−2	10.01 ± 0.67 ^1^
**7 b**	−2.03 ± 0.06	−2	1.00 ± 0.10 ^2^
**7 c**	−2.51 ± 0.09	−2	2338.00 ± 116.83 ^3^
**8** ^4^	−1.39 ± 0.04	−3	-
**8 a**	−1.44 ± 0.01	−3	15.09 ± 1.04 ^1^
**8 b**	−1.66 ± 0.03	−3	1.58 ± 0.04 ^2^
**8 c**	−2.17 ± 0.14	−3	3893.50 ± 295.89 ^3^
**9** ^4^	−1.29 ± 0.11	−4	-
**9 a**	−1.45 ± 0.06	−4	58.27 ± 3.66 ^1^
**9 b**	−1.42 ± 0.13	−4	5.73 ± 0.50 ^2^
**9 c**	−1.97 ± 0.18	−4	7425.75 ± 207.44 ^3^
**10** ^4^	−3.35 ± 0.15	−3	-
**10 a**	−3.78 ± 0.04	−3	19.13 ± 1.44 ^1^
**10 b**	−3.36 ± 0.02	−3	3.98 ± 0.07 ^2^
**10 c**	−4.06 ± 0.10	−3	4560.67 ± 436.66 ^3^

Competitive displacement studies were performed on ^1^ GRPR-expressing HEK, ^2^ MC1R-expressing B16F10 and ^3^ integrin α_v_β_3_-expressing U87MG cells. ^4^ Values were taken from [20,21].

## Data Availability

Data is contained within the article and Supplementary Materials.

## Supplementary Materials

The following data are available online at: https://www.mdpi.com/article/10.3390/ph14100989/s1. HPLC, ESI and MALDI mass spectra and normalized absorption and emission spectra of **7 a**-**10 a**, **7 b**-**10 b** and **7 c-10 c**. Figure S1: Analytical data for **7 a** (HPLC, ESI and MALDI mass spectroscopy); Figure S2: Analytical data for **8 a** (HPLC, ESI and MALDI mass spectroscopy); Figure S3: Analytical data for **9 a** (HPLC, ESI and MALDI mass spectroscopy); Figure S4: Analytical data for **10 a** (HPLC, ESI and MALDI mass spectroscopy); Figure S5: Analytical data for **7 b** (HPLC, ESI and MALDI mass spectroscopy); Figure S6: Analytical data for **8 b** (HPLC, ESI and MALDI mass spectroscopy); Figure S7: Analytical data for **9 b** (HPLC, ESI and MALDI mass spectroscopy); Figure S8: Analytical data for **10 b** (HPLC, ESI and MALDI mass spectroscopy); Figure S9: Analytical data for **7 c** (HPLC, ESI and MALDI mass spectroscopy)); Figure S10: Analytical data for **8 c** (HPLC, ESI and MALDI mass spectroscopy); Figure S11: Analytical data for **9 c** (HPLC, ESI and MALDI mass spectroscopy); Figure S12: Analytical data for **10 c** (HPLC, ESI and MALDI mass spectroscopy); Figure S13: Normalized absorption and emission spectra of **7 a** (top left), **7 b** (top right) and **7 c** (bottom left), recorded in H_2_O at a concentration of c = 1.0 × 10^−5^ mol/L, emission wavelength λ = 400 nm; Figure S14: Normalized absorption and emission spectra of **8 a** (top left), **8 b** (top right) and **8 c** (bottom left), recorded in H_2_O at a concentration of c = 1.0 × 10^−5^ mol/L, emission wavelength λ = 400 nm; Figure S15: Normalized absorption and emission spectra of **9 a** (top left), **9 b** (top right) and **9 c** (bottom left), recorded in H_2_O at a concentration of c = 1.0 × 10^−5^ mol/L, emission wavelength λ = 700 nm; Figure S16: Normalized absorption spectra of **10 a** (top left), **10 b** (top right) and **10 c** (bottom left), recorded in H_2_O at a concentration of c = 1.0 × 10^−5^ mol/L, emission wavelength λ = 400 nm.
