# Supplementary material for: Synthesis, Characterization and In Vitro Evaluation of Hybrid Monomeric Peptides Suited for Multimodal Imaging by PET/OI: Extending the Concept of Charge—Cell Binding Correlation
Source: Pharmaceuticals (Basel). 2021 Sep 28;14(10):989. doi: 10.3390/ph14100989 (PMC8541144; doi:10.3390/ph14100989)
Supplement: Supplementary file 1 [file pharmaceuticals-14-00989-s001.zip › pharmaceuticals-1390762-supplementary.pdf]

Synthesis, Characterization and In Vitro Evaluation of Hybrid Monomeric Peptides Suited for Multimodal Imaging by PET/OI: Extending the Concept of Charge-Cell Binding Correlation

Marco Maspero[a,b], Xia Cheng[c], Valeska von Kiedrowski[c], Clelia Dallanoce[b], Björn Wängler[c], Ralph Hübner[a,†]* and Carmen Wängler[a,†]*

[a] Biomedical Chemistry, Department of Clinical Radiology and Nuclear Medicine, Medical Faculty Mannheim, Heidelberg University, Theodor-Kutzer-Ufer 1-3, 68167 Mannheim, Germany. E-mail: Ralph.Huebner@medma.uni-heidelberg.de (R.H.); Carmen.Waengler@medma.uni-heidelberg.de (C.W.)

[b] Medicinal Chemistry Section "Pietro Pratesi", Department of Pharmaceutical Sciences, University of Milan, Via L. Mangiagalli 25, 20133, Milan, Italy

[c] Molecular Imaging and Radiochemistry, Department of Clinical Radiology and Nuclear Medicine, Medical Faculty Mannheim, Heidelberg University, Theodor-Kutzer-Ufer 1-3, 68167 Mannheim, Germany

[†] Authors share last position

Table of Contents

Supplementary figures………..…………….. S2 – S7

Figures S1–S12. HPLC, ESI and MALDI mass spectra of **7** – **10 a**, **7** – **10 b** and **7** – **10 c**

Figures S13–S16 Normalized absorption and emission spectra of **7** – **10 a**, **7** – **10 b** and **7** – **10 c**

Supplementary figures

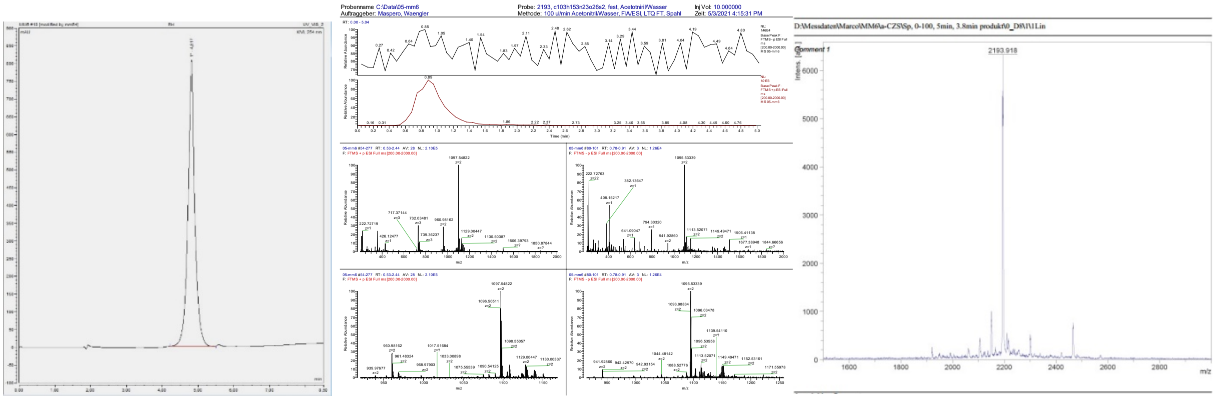

Figure S1 Analytical data for **7 a** (HPLC, ESI and MALDI mass spectroscopy).

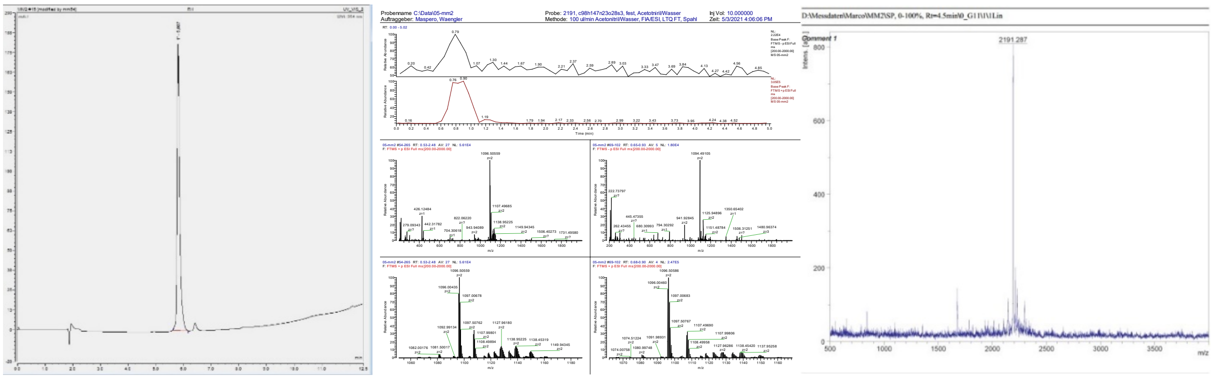

Figure S2 Analytical data for **8 a** (HPLC, ESI and MALDI mass spectroscopy).

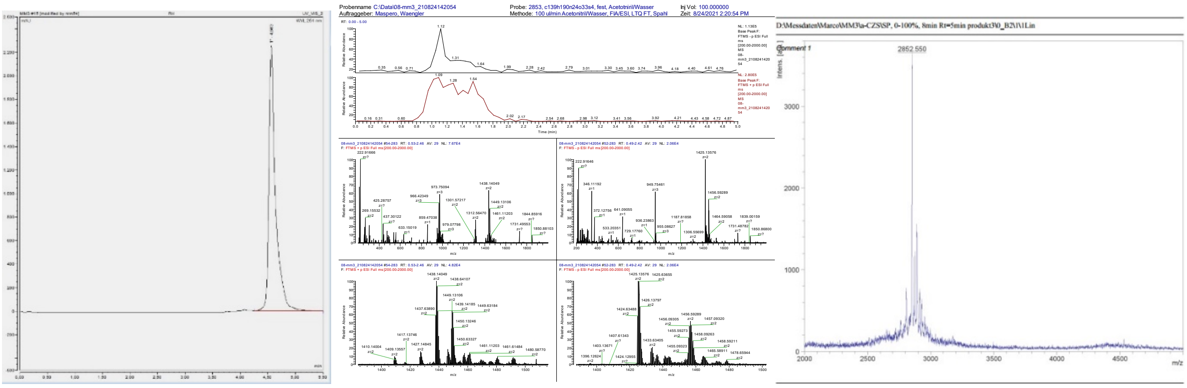

Figure S3 Analytical data for **9 a** (HPLC, ESI and MALDI mass spectroscopy).

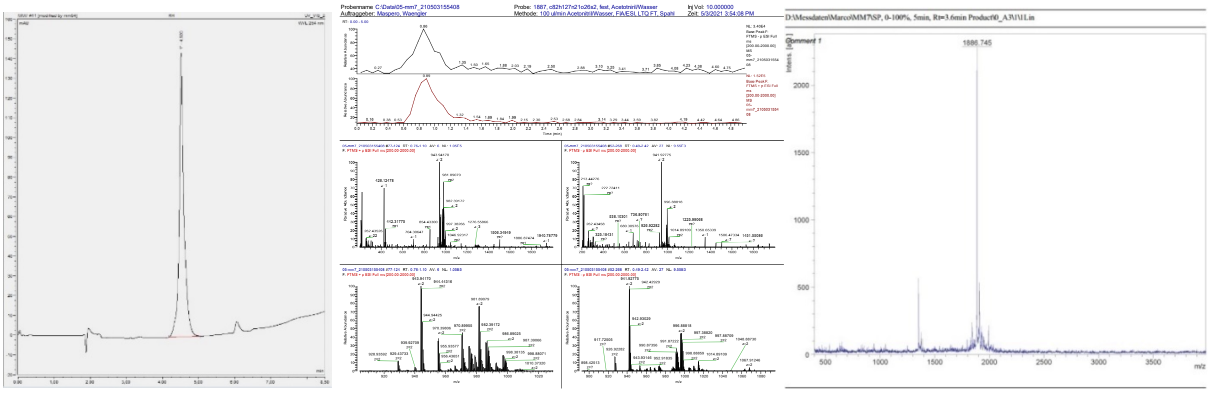

Figure S4 Analytical data for **10 a** (HPLC, ESI and MALDI mass spectroscopy).

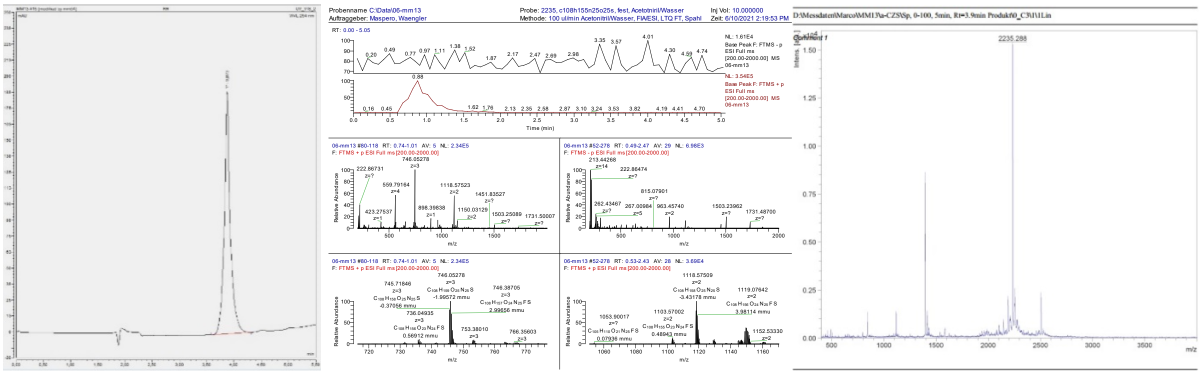

Figure S5 Analytical data for **7 b** (HPLC, ESI and MALDI mass spectroscopy).

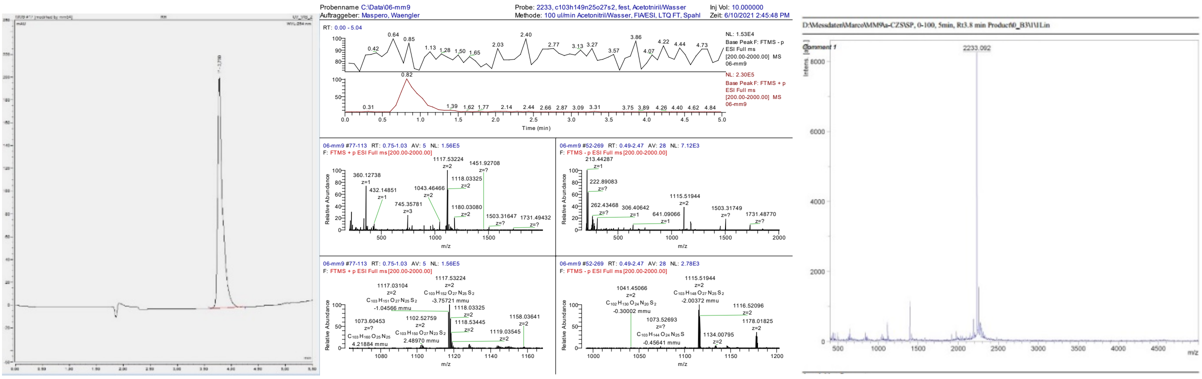

Figure S6 Analytical data for **8 b** (HPLC, ESI and MALDI mass spectroscopy).

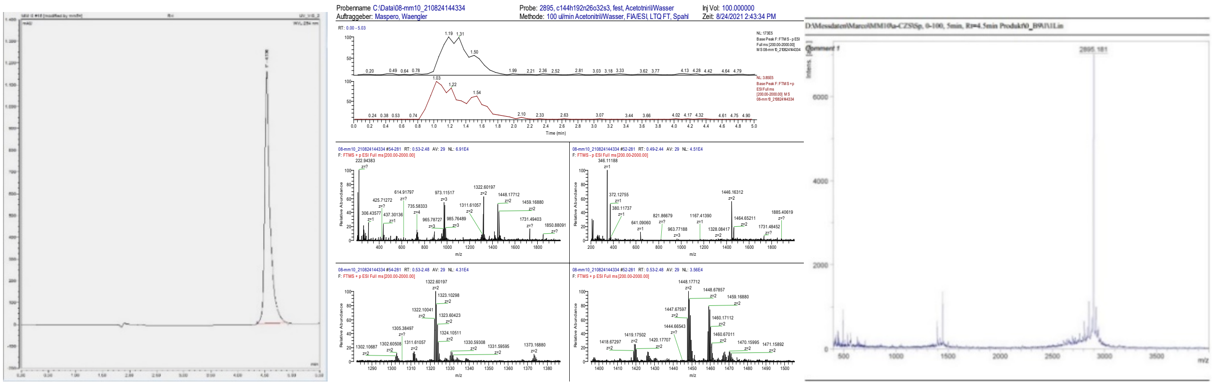

Figure S7 Analytical data for **9 b** (HPLC, ESI and MALDI mass spectroscopy).

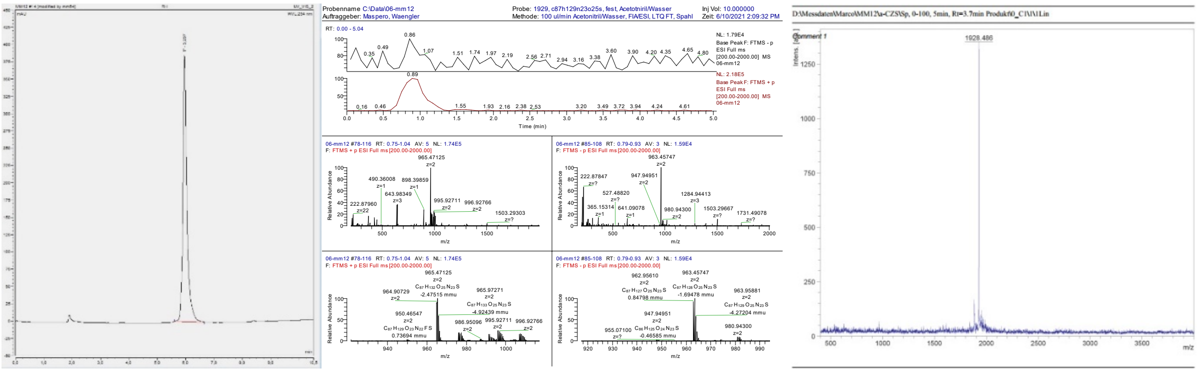

Figure S8 Analytical data for **10 b** (HPLC, ESI and MALDI mass spectroscopy).

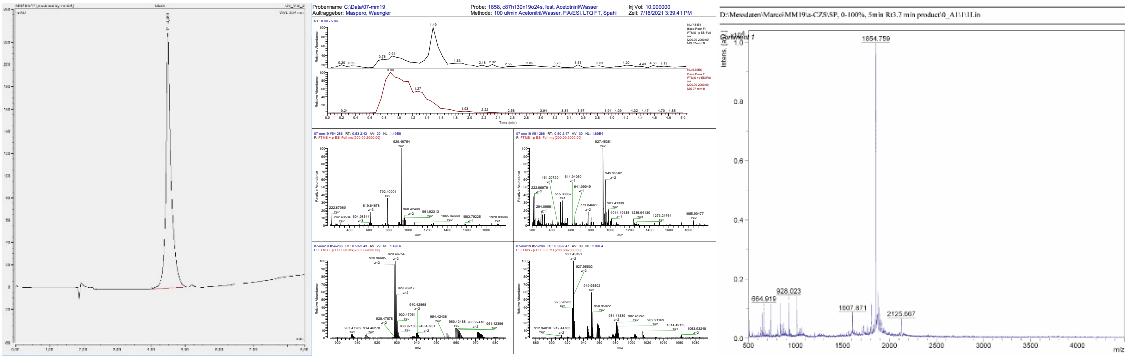

Figure S9 Analytical data for **7 c** (HPLC, ESI and MALDI mass spectroscopy).

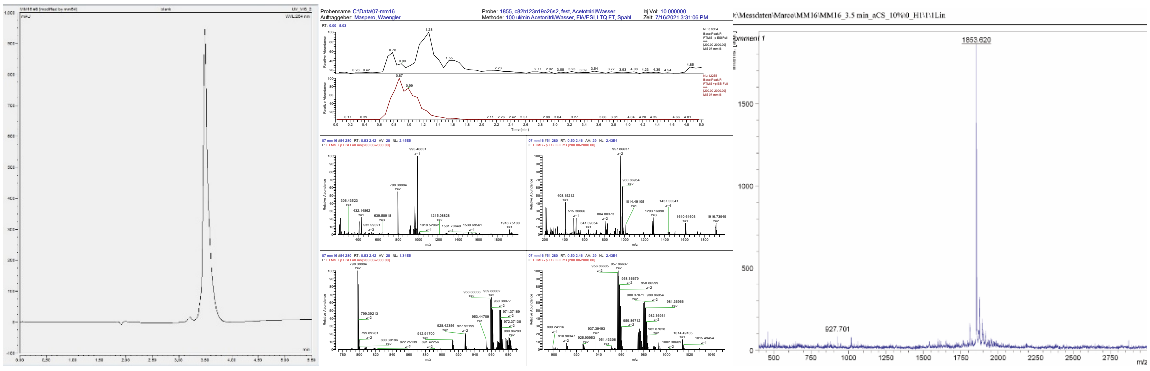

Figure S10 Analytical data for **8 c** (HPLC, ESI and MALDI mass spectroscopy).

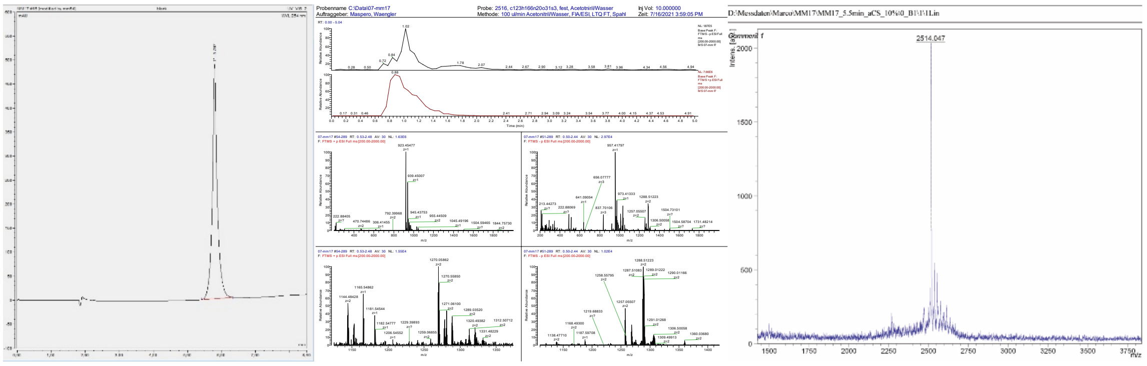

Figure S11 Analytical data for **9 c** (HPLC, ESI and MALDI mass spectroscopy).

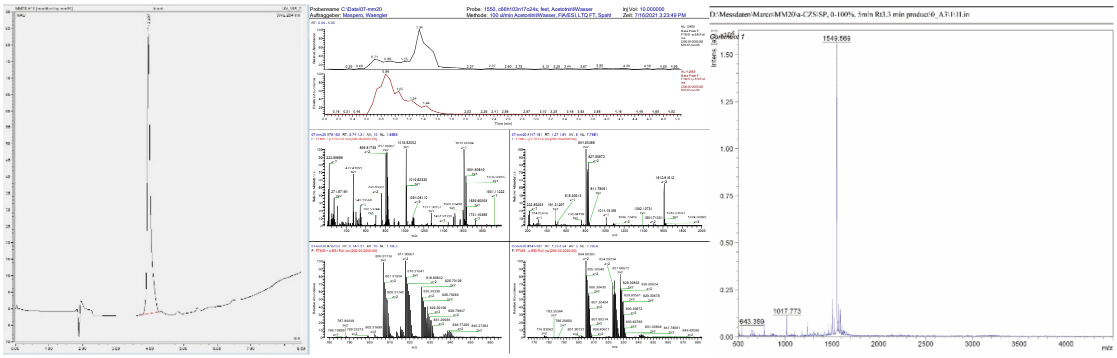

Figure S12 Analytical data for **10 c** (HPLC, ESI and MALDI mass spectroscopy).

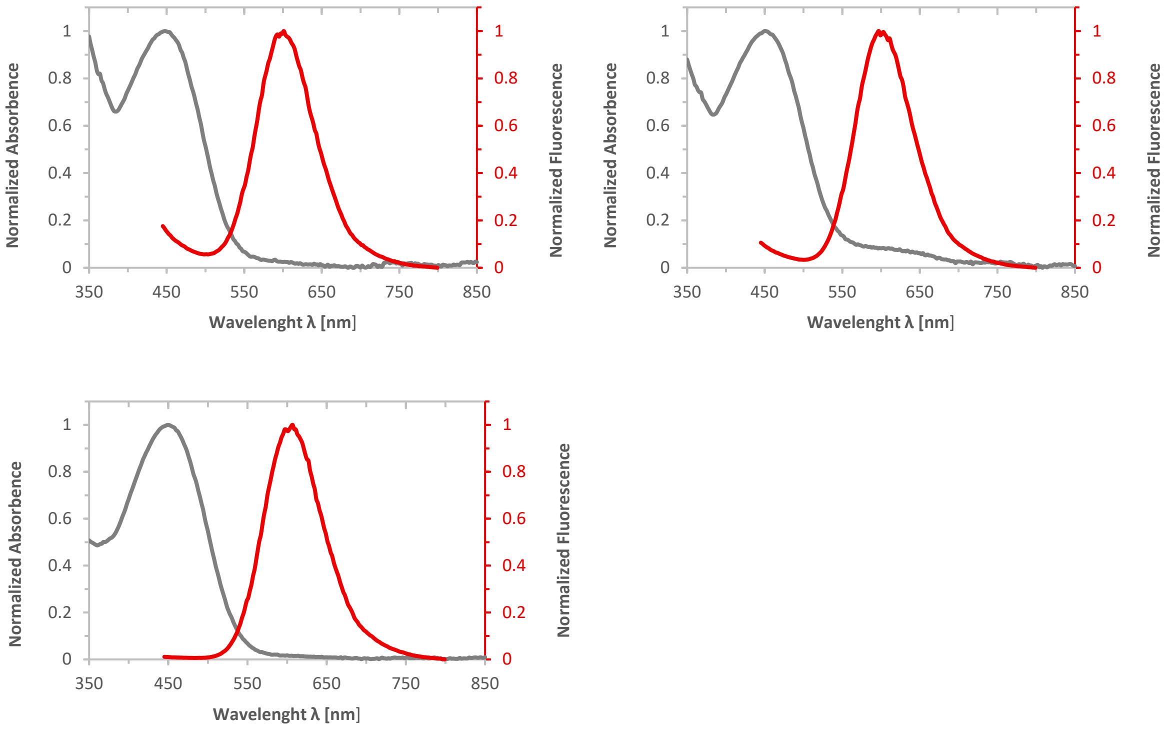

Figure S13 Normalized absorption and emission spectra of **7 a** (top left), **7 b** (top right) and **7 c** (bottom left), recorded in H_2O at a concentration of c = 1.0 x 10^{-5} mol/L, emission wavelength λ = 400 nm.

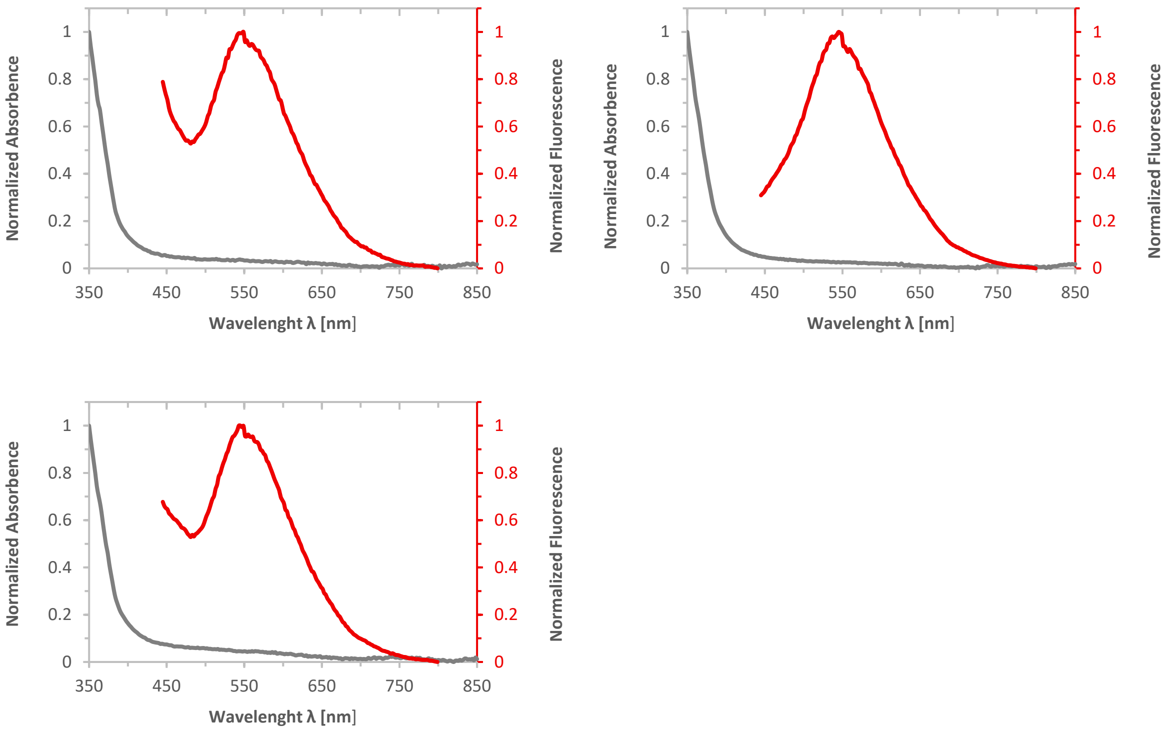

Figure S14 Normalized absorption and emission spectra of **8 a** (top left), **8 b** (top right) and **8 c** (bottom left), recorded in H_2O at a concentration of c = 1.0 x 10^{-5} mol/L, emission wavelength λ = 400 nm.

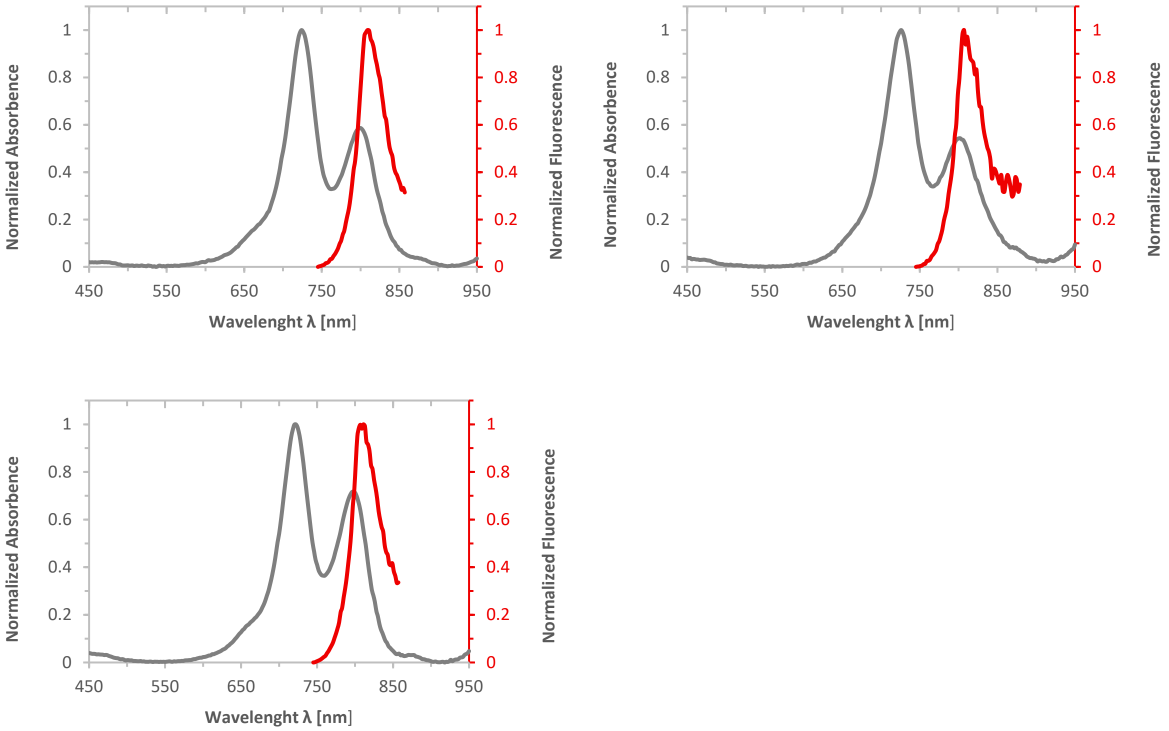

Figure S15 Normalized absorption and emission spectra of **9 a** (top left), **9 b** (top right) and **9 c** (bottom left), recorded in H_2O at a concentration of c = 1.0 x 10^{-5} mol/L, emission wavelength λ = 700 nm.

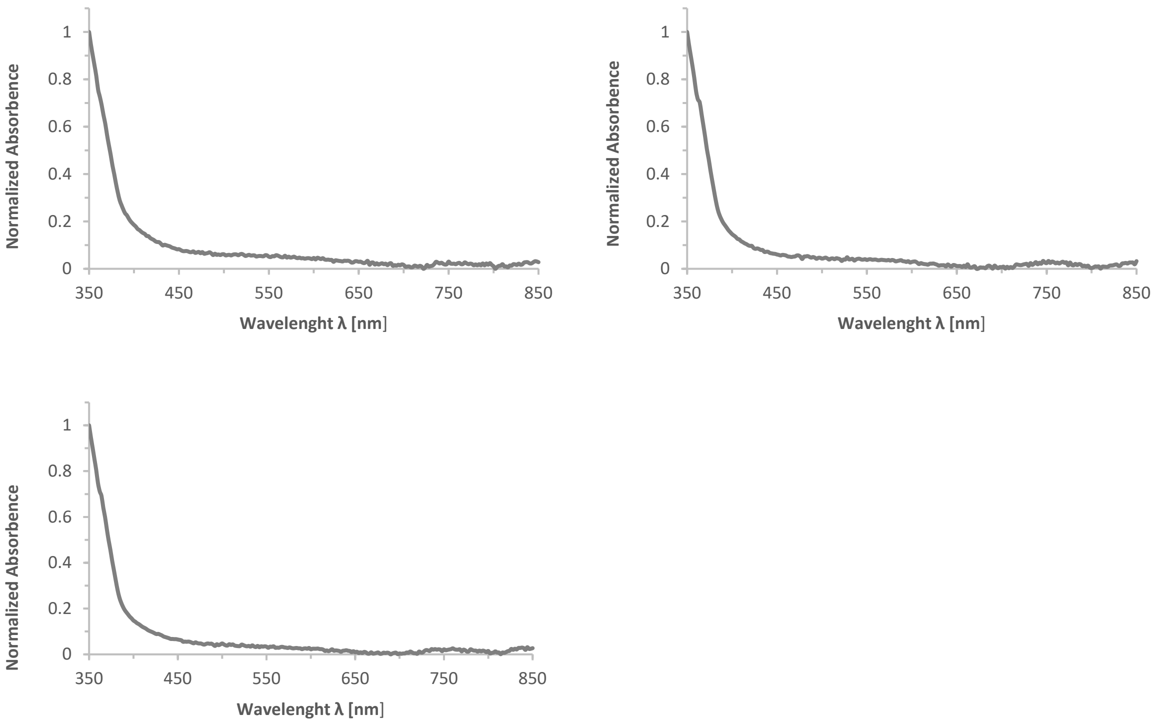

Figure S16 Normalized absorption spectra of **10 a** (top left), **10 b** (top right) and **10 c** (bottom left), recorded in H_2O at a concentration of c = 1.0 x 10^{-5} mol/L, emission wavelength λ = 400 nm.
